# MicroRNA Binding Site Polymorphisms in Hepatocellular Carcinoma: Implications for Pathogenesis, Prognosis, and Therapeutic Response

**DOI:** 10.1155/ijog/9520856

**Published:** 2025-12-15

**Authors:** Sara Rezapour, Farin Khodam Mohammadi, Mahmoud Darweesh, Saeed Mohammadi, Saeid Afshar

**Affiliations:** ^1^ Cancer Research Center, Institute of Cancer, Hamadan University of Medical Sciences, Hamadan, Iran, umsha.ac.ir; ^2^ Clinical Research Development Unit of Besat Hospital, Hamadan University of Medical Science, Hamadan, Iran, umsha.ac.ir; ^3^ Immunology Laboratory, Natural and Medical Sciences Research Center, University of Nizwa, Nizwa, Oman, unizwa.edu.om; ^4^ Department of Microbiology and Immunology, Al-Azhar University, Assiut, Egypt, azhar.edu.eg; ^5^ Golestan Research Center of Gastroenterology and Hepatology, Golestan University of Medical Sciences, Gorgan, Iran, goums.ac.ir; ^6^ Department of Medical Biotechnology, School of Advanced Medical Sciences and Technologies, Hamadan University of Medical Sciences, Hamadan, Iran, umsha.ac.ir

**Keywords:** angiogenesis, apoptosis, biomarkers, DNA repair, hepatocellular carcinoma, immune regulation, liquid biopsy, microRNAs, precision medicine, single nucleotide polymorphisms

## Abstract

Hepatocellular carcinoma (HCC) is a major global health challenge, characterized by complex molecular mechanisms. This review focuses on the crucial roles of microRNAs (miRNAs) in HCC development, progression, and therapeutic response. The regulation of gene expression and several critical cellular processes is carried out by miRNAs. These small, noncoding RNAs play a significant role in apoptosis, DNA repair, immune regulation, angiogenesis, cell migration, invasion, and tumor progression. MiRNAs have been identified as valuable noninvasive biomarkers, which suggests their potential use in early diagnosis, prognosis, and tracking the effectiveness of treatments. The relationship between single nucleotide polymorphisms (SNPs) in miRNA binding sites and their impact on both the vulnerability to and the development of HCC is also a topic of this discussion. These genetic variations can alter miRNA‐mRNA interactions, affecting the expression of critical genes involved in HCC, which modulates key cellular processes such as apoptosis, DNA repair, and immune regulation. Emerging technologies like liquid biopsies and exosomal miRNA analysis are explored for their potential to revolutionize HCC diagnosis and treatment. This first‐of‐its‐kind comprehensive review consolidates current findings on miRNA‐SNP interactions across four major HCC pathogenic pillars (apoptosis, DNA repair, immune evasion, and metastasis), providing novel, noninvasive genetic biomarkers for HCC risk stratification, prognosis prediction, and tailoring individualized therapeutic regimens.

## 1. Introduction

### 1.1. Hepatocellular Carcinoma: Global Burden and Pathogenesis

The global burden of liver cancer is significant, as it is the fifth most common malignancy and responsible for the fourth highest number of cancer‐related deaths. The highest rates of this disease are found in East Asia and sub‐Saharan Africa [1]. Among men, it is the fourth most diagnosed cancer and second deadliest [2]. Hepatocellular carcinoma (HCC), arising from hepatocytes, accounts for over 80% of primary liver cancers, while intrahepatic cholangiocarcinoma (ICC) originates in the bile ducts [3]. Secondary liver metastases, often from colorectal cancer, further complicate the disease burden [4].

HCC develops through a multistep process driven by genetic and environmental factors, with cirrhosis as a common precursor [5]. Chronic liver injury, triggered by hepatitis B/C viruses (HBV/HCV), alcohol, aflatoxins, obesity, type 2 diabetes, or smoking, creates a pro‐carcinogenic microenvironment [2]. Molecular alterations in a cell can include mutations in tumor suppressor genes (such as TP53, PTEN) and proto‐oncogenes (such as RAS, MYC), chromosomal instability, and activation of inflammatory pathways (including, IL‐6/STAT3, NF‐*κ*B) [6–8]. Inflammatory cytokines, particularly IL‐6 and TNF‐*α*, activate critical signaling pathways including STAT3 and NF‐*κ*B, promoting hepatocyte proliferation, fibrogenesis, and neoplastic transformation. HCC pathogenesis involves dysregulation of key molecular pathways such as JAK/STAT, Ras/Raf/MAPK, PI3K/AKT/mTOR, and ubiquitin–proteasome systems, which control cellular proliferation, survival, and apoptotic processes [7, 9]. Furthermore, VEGF‐mediated angiogenesis is crucial in HCC progression and metastatic dissemination, with elevated VEGF expression correlating strongly with adverse clinical outcomes [7, 10]. Furthermore, the pathogenesis of HCC can be influenced by factors such as viral infections, conditions like nonalcoholic fatty liver disease (NAFLD), and oxidative stress [11]. These chronic insults are known to induce specific changes in the cellular miRNome, directly linking environmental factors to post‐transcriptional dysregulation.

### 1.2. Diagnostic Landscape and the Crucial Role of MicroRNAs

Current diagnostic approaches for HCC focus on early detection, crucial for improving outcomes [12, 13]. Diagnosis using conventional methods relies on imaging techniques, including ultrasound, computed tomography (CT), and magnetic resonance imaging (MRI) [14]. Ultrasound is the standard HCC screening tool due to its accessibility and low cost, though less sensitive for small tumors. Advanced imaging (CT/MRI) improves detection but is prohibitively expensive for routine use [12, 15, 16].

Serological biomarkers, including alpha‐fetoprotein (AFP), are still the main diagnostic tools for HCC, though their sensitivity and specificity are inadequate, especially for early stages [14, 17]. Several alternatives, such as AFP‐L3 and des‐*γ*‐carboxy prothrombin (DCP), have been presented, but their diagnostic accuracy is still insufficient [14, 18]. Recent studies also introduced potential diagnostic markers, including Glypican‐3 (GPC3), Golgi protein‐73 (GP73) [13], and microRNAs (including, miR‐122, miR‐21) [19]. Emerging molecular technologies, including liquid biopsies analyzing cell‐free DNA (cfDNA), circulating tumor cells (CTCs), and extracellular vehicles (EVs), offer noninvasive options with high promise for early detection, by capturing tumor‐specific genetic and epigenetic changes [14, 20]. In particular, cfDNA methylation patterns and miRNA signatures in EVs are considered highly promising for improving early detection [21–23]. Despite these advances, challenges in cost, standardization, and validation limit their integration into routine practice [12, 15, 16, 20]. These gaps highlight the importance of developing more specific biomarkers and utilizing molecular advances to enhance HCC early diagnostic and its accuracy [18, 23–25].

**Figure 1:**
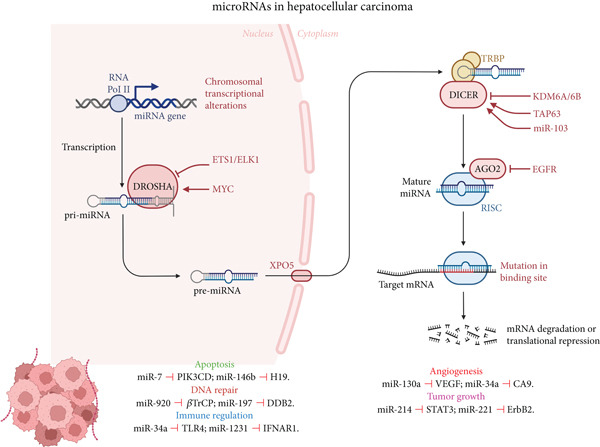
Schematic representation of microRNA (miRNA) biogenesis and function, and the impact of chromosomal/transcriptional alterations and mutations in miRNA binding sites in the context of hepatocellular carcinoma (HCC). The biogenesis pathway begins with the transcription of miRNA genes by RNA polymerase II, resulting in primary miRNA (pri‐miRNA). Pri‐miRNA is processed by the Drosha complex in the nucleus to form precursor miRNA (pre‐miRNA), which is then exported to the cytoplasm by XPO5. In the cytoplasm, pre‐miRNA is cleaved by Dicer to generate mature miRNA. Mature miRNA is loaded into the RNA‐induced silencing complex (RISC), where it guides the complex to its target messenger RNA (mRNA) based on sequence complementarity. This interaction can lead to mRNA degradation or translational repression, thereby regulating gene expression. The figure also highlights examples of miRNAs and their target genes involved in key processes dysregulated in HCC, including apoptosis (miR‐7 targeting PIK3CD, miR‐146b targeting H19), DNA repair (miR‐920 targeting *β*TrCP, miR‐197 targeting DDB2), immune regulation (miR‐34a targeting TLR4, miR‐1231 targeting IFNAR1), angiogenesis (miR‐130a targeting VEGF, miR‐34a targeting CA9), and tumor growth (miR‐214 targeting STAT3, miR‐221 targeting ErbB2). Mutations in the miRNA binding sites of target mRNAs can disrupt this regulatory process, contributing to HCC pathogenesis. This is an original figure created by the authors using BioRender (license obtained).

MicroRNAs (miRNAs) are short, noncoding RNAs crucial for post‐transcriptional regulation. By binding to specific mRNAs, often within the 3’‐untranslated regions, miRNAs control vital cell processes such as differentiation, proliferation, and apoptosis. There is a complex regulatory network between miRNAs and mRNAs. One miRNA can control the expression of numerous mRNAs, and in turn, a single mRNA can be a target for multiple miRNAs [26]. This interaction typically results in translational inhibition or mRNA degradation, leading to reduced protein expression [27]. miRNAs are categorized as either oncomiRs, which promote tumor growth, or tumor suppressors, which inhibit tumor development and progression [26]. In HCC, dysregulation of miRNAs significantly contributes to tumorigenesis, progression [28, 29], and response to treatment [30]. For example, miR‐122, a liver‐specific miRNA, is downregulated in HCC and associated with tumor growth and poor prognosis. HCC shows an increase in the oncogenic miRNA miR‐21, which drives cell proliferation and invasion. In contrast, miR‐199a‐3p is downregulated in HCC and is linked to angiogenesis, proliferation, and apoptosis [29]. Furthermore, exosomal miRNAs are being explored as noninvasive biomarkers for HCC detection [31]. Given their role in regulating gene expression and their involvement in various stages of HCC progression [19, 32], miRNAs and their binding sites are potential noninvasive biomarkers for the early detection, prognosis, and monitoring of treatment response in HCC [29, 32, 33]. Specifically, they offer a less invasive alternative to tissue biopsies by enabling the identification of genetic alterations in circulating exosomes or cell‐free RNA. Figure 1 demonstrates the schematic representation of microRNA‐SNP interaction and their involvement in HCC pathogenesis.

Recognizing the crucial role of miRNAs in HCC and the potential impact of SNPs on their function, this review aims to provide a first‐of‐its‐kind, comprehensive synthesis of miRNA‐SNP interactions across these four pathogenic pillars, thereby establishing a new molecular framework for HCC risk stratification, prognosis, and the design of targeted therapeutic strategies.

## 2. miRNA‐Mediated Regulation of Apoptosis in HCC and the Impact of SNPs

Apoptosis, a programmed cell death process, can function as a protective mechanism in cells, but it also paradoxically occurs alongside the progression and proliferation of tumor cells [26]. Recent research has revealed that miRNAs are involved in both the extrinsic and intrinsic pathways of apoptosis [34]. miRNAs are classified into two groups: pro‐apoptotic, which promote cell death, and anti‐apoptotic, which inhibit it. Pro‐apoptotic miRNAs can suppress cancer development, whereas anti‐apoptotic miRNAs can facilitate it [26]. Furthermore, transcripts related to autophagy and apoptosis can indirectly influence each other by competing for common miRNA binding sites [35]. According to recent research, the presence of SNPs within the miRNA binding sites of specific genes, including *BCL-xl*, *H19*, *PIK3CD*, and *FOXO1*, impacts the process of apoptosis [6, 19, 22, 30] (Table 1).

**Table 1:** miRNA SNPs associated with apoptosis and DNA damage response in HCC.

**miRNA name**	**SNP**	**Biological function**	**Target genes**	**Genetic pathway**	**Clinical relevance**
miR‐146b‐3p	rs3741219	Pro‐apoptotic, promotes apoptosis and silences H19 expression.	H19, TRAF6	p53	Associated with shortened survival in HCC patients.
miR‐1539	rs3741219	Pro‐apoptotic, down regulates H19 expression via SNP modification.	H19	p53	Improves survival outcomes in HCC by suppressing H19 expression.
miR‐7	—	Pro‐apoptotic, inhibits tumorigenesis by targeting PIK3CD and mTOR.	PIK3CD, mTOR, p70S6K	PI3K/AKT	Prevents metastasis, arrests cell cycle, and suppresses migration.
let‐7b	rs3208684	Pro‐apoptotic, suppresses oncogenes like RAS and Bcl‐xl, involved in apoptosis regulation.	Bcl‐xl, RAS, HMGA, c‐Myc, cyclin‐D	Ras/MAPK	SNP rs3208684 disrupts let‐7b binding to Bcl‐xl, increasing 5‐FU sensitivity in HCC cells.
miR‐137	rs17592236	Pro‐apoptotic, regulates FOXO1 expression and modulates apoptosis via SNP rs17592236.	FOXO1	PI3K‐Akt‐FOXO1	Decreases hereditary HCC susceptibility by affecting FOXO1 expression.
miR‐920	rs16405	Regulates DNA repair via *β*TrCP, affects WNT signaling, indirectly involved in cell cycle regulation.	*β*TrCP, PLK1	WNT	SNP rs16405 disrupts miR‐920 binding to *β*TrCP, leading to tumor development and HCC susceptibility.
miR‐197	rs1050244	Pro‐apoptotic, activates BAD, BAX, BID, BIM, impacts response to DNA damage.	DDB2	p53/p21	SNP rs1050244 disrupts miR‐197 binding, leading to DDB2 up regulation and reduced HCC susceptibility.
miR‐133a	rs1050244	Tumor suppressor, modulates p53/p21 pathway, involved in NER activity.	DDB2	p53/p21	SNP rs1050244 disrupts miR‐133a binding, increasing DDB2 expression and decreasing HCC risk.
miR‐129‐3p	rs12593359	Regulates calcium signaling, mitochondrial function, ROS, and DNA damage repair.	RAD51	Homologous recombination	SNP rs12593359 disrupts miR‐129‐3p binding to RAD51, inhibiting invasion and metastasis in HCC.
let‐7	rs7963551	Tumor suppressor, regulates cell signaling pathways, impacts RAD52 function.	RAD52	Homologous recombination	SNP rs7963551 reduces miR‐let‐7 binding, increasing RAD52 expression, associated with HBV‐related HCC risk.

**miR-146b** is located at 10q24.32 [36, 37], is involved in the innate immune response to oncogenic transformation, and is implicated in cancer [38]. Specifically, miR‐146b‐5p promotes apoptosis [39], and its expression levels have been linked to HCC [40]. According to research by Yemei Song et al., miR‐146b‐3p is linked to poor survival in HCC patients. The study demonstrated that this miRNA significantly decreases the expression of *H19* at the rs3741219 site [41]. *H19* expression may induce cell apoptosis via the p53 protein [42]. Song et al. also found a significant association between the *H19* SNP rs3741219 and overall survival in HCC [42].

**miR-1539**, consisting of 21 nucleotides, has been shown to induce angiogenesis and promote endothelial cell survival during ontogenesis. The functional rs3741219 SNP, located in the 3’‐region of *H19*, causes allelic downregulation of the *H19* gene by creating a binding site for both miR‐146b‐3p and miR‐1539 [40]. The *H19* gene is involved in a number of biological functions, including cell proliferation, apoptosis, differentiation, and autophagy. It also plays a role in the development of various diseases, including tumors [43]. The rs3741219 SNP in the *H19* gene may increase target sites for miR‐1539. Song et al. suggested that miR‐1539 downregulation of *H19* expression significantly contributes to HCC survival [44].

**miR-7** is a powerful miRNA involved in numerous signaling pathways [45] and is encoded by three separate genes [46]. In mammals, miR‐7 primarily acts as a tumor suppressor and helping to regulate important cellular functions, including proliferation, differentiation, and apoptosis [45]. Fang et al. showed that *mTOR* and *p70S6K*, key downstream signals of *PI3K*, possess miR‐7 target sites in their 3’UTR [19, 47]. *PI3K* signaling is important for survival, apoptosis, proliferation, migration, and invasion in various cancer types [48]. Fang et al. reported that miR‐7 prevents tumorigenesis and metastasis in HCC by targeting a new site. Overexpression of *PIK3CD* and miR‐7 disrupts cancer cell migration through cell cycle arrest [19, 47].

**miR-let7b** is among the first microRNAs discovered and is located on chromosomes 9, 22, and X [49]. Most let‐7 regulatory proteins have been extensively studied in relation to development, proliferation, differentiation, and cancer. Previous research has shown that this microRNA can regulate several key oncogenes, including *RAS*, *HMGA*, *c-Myc*, and *cyclin-D*, leading to the inhibition of cancer development, maturation, and progression [50]. The 3’‐UTR of the *Bcl-xl* gene may contain a potential miRNA binding site for let‐7b [51]. The Bcl‐2 family of proteins is crucial for controlling apoptosis [52], and changes in the expression of *BCL-2* family members have been recognized as vital in cancer development and progression [53]. The rs3208684 SNP disrupts the binding of miR‐let‐7b to the 3’UTR of *Bcl-xl*, leading to the increased expression of this gene, effectively suppressing or disrupting the binding of let‐7b to its target and subsequently improving sensitivity to 5‐FU through downregulation of *Bcl-xl* in HCC [52, 54].

**miR-137** is placed on chromosome 1p21.3 [55] and impacts crucial cellular functions such as apoptosis, playing a major role as a tumor suppressor in various cancer types [56]. The 2015 study by Tan and Chao et al. showed that the rs17592236 SNP can decrease the risk of HCC by modifying the binding affinity of miR‐137 to the *FOXO1* 3’UTR [57]. The transcription factor FOXO1 regulates cell cycle, apoptosis, and oxidative stress [58], and FoxOs control the proteins that stimulate apoptosis [59]. Tan and Chao et al.’s research showed that miR‐137 can influence *FOXO1* expression by interacting with the rs17592236 polymorphic site. This interaction influences the development of HCC and apoptosis through the PI3K‐Akt‐FOXO1 pathway [57].

While the accumulating data robustly link specific miRNA‐SNPs to altered apoptotic pathways in HCC, a critical assessment of the literature reveals several common methodological limitations. Many published association studies suffer from small sample sizes, lack of functional validation, or significant population heterogeneity, which limits the generalizability and mechanistic certainty of their findings. For instance, SNP–outcome associations often vary across different ethnic cohorts, necessitating large‐scale, multi‐ethnic meta‐analyses. Furthermore, the mechanistic interpretations derived solely from in silico predictions should be rigorously validated. Future research should prioritize robust experimental validation using techniques such as luciferase reporter assays to confirm the direct impact of the SNP on miRNA–mRNA binding affinity, alongside CRISPR/Cas9‐mediated gene editing and functional knockdown models in HCC cell lines to precisely define the downstream biological consequences.

## 3. MicroRNAs and Their SNPs in DNA Damage Response (DDR) Genes

The human genome constantly faces a barrage of endogenous and exogenous genotoxic insults, leading to numerous DNA damage events daily [60]. This constant assault leads to numerous DNA damage events every day. To protect against these threats and maintain the integrity of the genome, a complex DNA damage response (DDR) network is activated. microRNAs have emerged as crucial regulators of the DDR, influencing key cellular events such as cell cycle control and DNA repair mechanisms [61]. SNPs located within the microRNA target regions of genes involved in DNA repair can modulate miRNA–mRNA interactions. These alterations can affect the expression levels of these critical DNA repair genes, potentially impacting an individual’s capacity for DNA repair and consequently influencing disease susceptibility, including the risk of developing various cancers [62]. Several studies have indicated the relationship between SNPs found within miRNA binding sites of genes such as *BTRC*, *DDB2*, *RAD51*, and *RAD52*, and their effects on DNA damage repair pathways [63–66] (Table 1).

**MicroRNA-920** (miR‐920) is a gene located on chromosome 12 [67] *that* has a predicted binding site within the 3’ untranslated region (3’UTR) of the *BTRC* gene. The *BTRC* gene produces *β*‐transducin repeat containing E3 ubiquitin protein ligase. This protein is a component of the Skp1‐Cullin‐F‐box (SCF) ubiquitin ligase complex that tags specific targets for proteasomal destruction, including Polo‐like kinase 1 (PLK1) [68]. PLK1 is a key regulator of cell cycle progression and has indirect involvement in DNA repair processes [64]. The SCF*β*TrCP complex ubiquitinates PLK1 during the G1 and S phases of the cell cycle, leading to its degradation [69]. A study by Chen et al. investigated the functional impact of the rs16405 polymorphism within the *BTRC* 3’UTR, which lies in the miR‐920 binding site [64]. Their findings indicated that the rs16405 alleles disrupted the binding affinity of miR‐920 to the *BTRC* mRNA. This impaired interaction led to an upregulation of *BTRC* expression and, through the modulation of WNT signaling, contributed to HCC development.

**MicroRNA-197** (miR‐197), transcribed from chromosome 1p13.3, exhibits significant dysregulation across a spectrum of diseases, notably various cancers [70]. Functionally, miR‐197 can promote apoptosis by activating pro‐apoptotic proteins such as BAD, BAX, BID, and BIM, which are central to the apoptotic pathway and influence a cell’s response to DNA damage [71]. Qiu et al. [65] explored the interplay between the *DDB2* SNP rs1050244 and the binding of miR‐133a and miR‐197. The *DDB2* gene encodes the damage‐specific DNA binding protein 2, a crucial component of the nucleotide excision repair (NER) pathway, responsible for recognizing and removing bulky DNA lesions [65]. Their study suggested that the rs1050244 variant disrupted the interaction of both miR‐133a and miR‐197 with the *DDB2* mRNA, leading to an upregulation of *DDB2* expression and a potentially reduced risk of HCC [65]. This observation highlights a scenario where a SNP in a miRNA target site can have a protective effect against cancer development by enhancing the expression of a key DNA repair gene. miR‐133a, located on chromosome 11q13.3, is a tumor suppressor miRNA involved in tumor initiation and progression and positively regulates the p53/p21 signaling pathway, further underscoring the complex interplay between miRNAs and DNA repair [72, 73]. Mutations in the *DDB2* gene are known to impair DDB2–DNA or DDB2–DDB1 complex formation, consequently compromising NER activity [74].

**MicroRNA-129-3p** (miR‐129‐3p), located on chromosome 11p11.2 [47], is often dysregulated in many cancer types [75]. It has been linked to the regulation of DNA damage and intracellular calcium signaling pathways [76]. The rs12593359 polymorphism is in the 3’UTR of the *RAD51* gene, within a predicted binding site for miR‐129 [66]. The *RAD51* gene is a central player in maintaining DNA fidelity by facilitating homologous recombination repair of DNA double‐strand breaks (DSBs) [77]. A more recent study by Qiu et al. [66] hypothesized that the rs12593359 SNP in the *RAD51* 3’UTR could interfere with miR‐129‐3p binding [77]. Their findings suggested that this disruption could lead to increased *RAD51* expression, potentially hindering the suppression of HCC invasion and metastasis normally mediated by miR‐129‐3p. This illustrates how a SNP can modulate miRNA‐mediated regulation of a critical DNA repair gene, influencing cancer progression.

**The *let-7* family** of microRNAs participates in the regulation of diverse cell signaling pathways [78], and is generally considered to function as tumor suppressors. Li et al. [63] investigated the rs7963551 polymorphism located within a *let-7* target site in the 3’UTR of the *RAD52* gene. The *RAD52* gene encodes a protein involved in DNA repair, particularly in single‐strand annealing and potentially in alternative double‐strand break repair pathways. Rad52 can establish physical connections with replication protein A (RPA), a single‐stranded DNA binding complex It has also been implicated in RNA bridging to facilitate DNA break synapsis and ligation, potentially utilizing RNA as a template for reverse transcription‐dependent DNA repair [79]. Li et al. concluded that the rs7963551 allele reduced the binding affinity of *let-7* miRNAs to the *RAD52* 3’UTR, resulting in increased *RAD52* gene expression. Notably, this SNP was markedly linked with an increased risk of hepatitis B virus (HBV)‐related HCC [63].

## 4. Modulation of Immune Homeostasis in HCC by MicroRNAs and Related SNPs

The interaction between the host immune system and cancer development is a critical determinant of disease progression. While efficient anti‐tumor immunity can effectively control and eliminate malignant cells, tumor‐associated inflammation can paradoxically foster cancer growth, invasion, and metastasis [80]. MicroRNAs are determinant in modulating host immune homeostasis [81]. Certain miRNAs can facilitate immune evasion by cancer cells through mechanisms such as reducing cancer cell immunogenicity and dampening anti‐tumor immune responses [82]. The significant role of miRNAs in regulating gene expression, particularly of inflammatory mediators, is well‐established. Consequently, SNPs within the 3’UTRs of immune‐related genes can disrupt miRNA binding, altering their regulatory efficacy and potentially influencing cancer susceptibility [83]. Previous investigations have implicated SNPs within the miRNA binding sites of genes such as *IFNAR1*, *TLR4*, *PDCD1* (*PD-1*), and *IL1A* in the context of HCC [84–87] (Table 2).

**Table 2:** miRNA SNPs associated with immune modulation and miRNA maturation in HCC.

**miRNA name**	**SNP**	**Biological function**	**Target genes**	**Genetic pathway**	**Clinical relevance**
miR‐1231	rs17875871	Downregulates IFNAR1, inhibits HBV replication, regulates immune response.	IFNAR1	IFN signaling	SNP rs17875871 in IFNAR1 3’ UTR disrupts miR‐1231 binding, increasing HCC susceptibility through immune modulation.
miR‐34a	rs1057317	Master regulator of tumor suppression, controls immune response, regulates TLR4.	TLR4	Innate immunity	SNP rs1057317 affects miR‐34a binding to TLR4, leading to hyper activation of inflammation and increased HCC risk.
miR‐4717	rs10204525	Regulates PD‐1 expression, enhances secretion of TNF‐*α* and IFN‐*γ*, impacts immune response.	PD1	PD‐1 pathway	SNP rs10204525 in PD1 3’ UTR alters miR‐4717 binding, affecting immune regulation and increasing susceptibility to chronic HBV infection and HCC.
miR‐122	rs3783553	Liver‐specific miRNA, regulates inflammatory cytokines, involved in HBV/HCV infection.	IL‐1A	Inflammatory response	SNP rs3783553 in IL1A 3’UTR disrupts miR‐122 binding, increasing IL‐1*α* expression and contributing to HCC vulnerability.
miR‐199‐3p	rs3803012	Regulates multiple signaling pathways in tumor development; involved in miRNA transport.	RAN	miRNA nuclear export	SNP rs3803012 in RAN 3’ UTR affects miRNA binding, altering miRNA processing and increasing HBV‐related HCC risk.
miR‐574‐3p	rs1057035	Key regulator in miRNA processing; affects DICER function.	DICER1	miRNA maturation	SNP rs1057035 in DICER1 3’ UTR influences miRNA binding, potentially affecting HCC susceptibility.

**MicroRNA-1231** (miR‐1231) exhibits downregulation in several cancer types [88]. Emerging evidence suggests an association between miR‐1231 and liver cancer risk, with a demonstrated role in inhibiting HBV replication by targeting its core mRNA [89]. A 2012 study by Zhou et al. identified the rs17875871 polymorphism within a putative miR‐1231 target sequence in the 3’UTR of the *IFNAR1* gene [86]. Type I interferons (IFNs), including IFN*α* and IFN*β*, exert anti‐tumor effects primarily indirectly by activating immune cells to mediate the elimination of cancerous cells [90]. Proteins induced by IFN signaling possess antiviral, antiproliferative, and immunomodulatory properties, acting through the interferon alpha/beta receptor (IFNAR) complex [91]. In their study, Zhou et al. proposed that the rs17875871 SNP resides within a functional binding site for miR‐1231. The presence of this SNP could potentially alter the binding affinity of miR‐1231 to the *IFNAR1* mRNA transcript, leading to dysregulation of *IFNAR1* expression. Negative regulation of *IFNAR1* by enhanced miR‐1231 binding, influenced by the SNP, could potentially impair downstream IFN signaling and increase susceptibility to chronic HBV infection, a major etiological factor for HCC development [86].

**MicroRNA-34a** (miR‐34a) is a well‐established tumor suppressor miRNA and is expressed in immune cells, where it regulates their function, development, and survival [92]. This miRNA exerts significant immunomodulatory effects by targeting over 30 genes involved in diverse cellular pathways [92]. A 2014 study by Jiang et al. studied the effects of the rs1057317 polymorphism on miR‐34a binding to *TLR4* mRNA and its association with HCC risk [84]. Toll‐like receptor 4 (TLR4) is a very important pattern recognition receptor of the innate immune system, and its hyperactivation can trigger the release of various pro‐inflammatory cytokines in the pathogenesis of several diseases [92]. TLRs, in general, are essential for the activation of both innate and adaptive immune responses [93]. The findings from Jiang et al.’s research indicated that the rs1057317 SNP, within the miR‐34a binding site in the *TLR4* gene, could significantly influence the progression of hepatocellular carcinoma. The SNP‐mediated alteration in miR‐34a binding and subsequent TLR4 expression could disrupt the delicate balance of immune signaling, contributing to chronic inflammation and promoting HCC development.

**MicroRNA-4717** (miR‐4717) is engaged in the pathogenesis of chronic HBV infection, as one of the most important causes of HCC [94]. Functionally, miR‐4717 can downregulate the production of PD‐1 mRNA by directly binding to its 3’UTR [95], subsequently leading to increased production of IFN‐*γ* and TNF‐*α* [96] . A 2015 study by Zhang et al. predicted that the rs10204525 polymorphism influences the binding of miR‐4717 to the *PDCD1* 3’UTR and consequently affects PD‐1 expression [87]. Their research suggested that miR‐4717 modulates PD‐1 expression by interacting with the 3’UTR of *PDCD1* mRNA, resulting in the modification of immune regulation and influencing vulnerability to chronic HBV infection and HCC [96]. The SNP‐mediated alteration in this interaction could disrupt the fine‐tuning of T cell exhaustion and inflammatory responses in the liver [87].

**MicroRNA-122** (miR‐122) is an abundant miRNA found primarily in the liver. It plays a significant role in various liver diseases, including infections caused by hepatitis C and hepatitis B viruses. [97]. Accordingly, miR‐122 can modulate host immune responses by affecting the production of inflammatory cytokines [98]. A 2009 study by Gao et al. identified the rs3783553 polymorphism in the 3’UTR of the *IL1A* gene, demonstrating its impact on miR‐122 binding and the subsequent regulation of interleukin 1 alpha (IL‐1*α*), and its association with HCC risk [85]. IL‐1*α* is a pleiotropic cytokine with both pro‐ and anti‐tumorigenic roles in different cancer contexts and has been shown to be upregulated in several tumor types [99]. Gao et al.’s research indicated that the rs3783553 SNP disrupts a binding site for miR‐122, leading to increased transcription of IL1A. Consequently, this polymorphism in IL1A may play a role in susceptibility to HCC by altering the local inflammatory milieu within the liver [85].

## 5. Modulation of Tumor Migration, Invasion, and Angiogenesis by MicroRNAs and SNPs

Angiogenesis, the natural process of forming new blood vessels, is controlled by a delicate balance between factors that promote it and those that inhibit it. In diseases like cancer, this process becomes dysregulated, with an increase in pro‐angiogenic factors that are essential for a tumor to grow, invade surrounding tissues, and spread [100, 101]. Cancer cells exhibit disruptions in the normal regulatory mechanisms governing cell migration, facilitating local invasion and the establishment of distant metastases [102]. By controlling the expression of genes linked to angiogenesis, endothelial cell proliferation, migration, and tube formation, miRNAs are key regulators of these processes [103]. They can influence both pro‐ and anti‐angiogenic signaling pathways [104]. Several studies highlighted the critical involvement of miRNA deregulation in promoting tumor cell invasion, migration, and subsequent angiogenesis [105]. For instance, recent studies have identified miRNA binding site polymorphisms in genes implicated in migration, invasion, and angiogenesis, such as *RASA1*, *COL1A2*, *CA9*, *CD147*, *VHL*, and *RYR3*, which can influence susceptibility to hepatocellular carcinoma (HCC) [101, 106–110] (Table 3).

**Table 3:** miRNA SNPs associated with tumor progression, invasion, and related processes in HCC.

**miRNA name**	**SNP**	**Biological function**	**Target genes**	**Genetic pathway**	**Clinical relevance**
miR‐let7c	rs6147150	Tumor suppressor, inhibits Ras/NF*κ*B signaling to reduce cancer stem cell properties.	ERBB4	Tumor growth inhibition	SNP rs6147150 in miR‐let7c seed region affects miR binding to ERBB4, influencing HCC progression.
miR‐3196	rs884225	Downregulates EGFR, inhibiting cell proliferation and tumor growth.	EGFR	Tumor growth inhibition	SNP rs884225 enhances miR‐3196 binding to EGFR, suppressing proliferation in HCC.
miR‐130a/miR‐19b	rs10474257	Regulates NF‐*κ*B signaling, VEGFA expression, cell invasion, migration, and angiogenesis.	RASA1	Angiogenesis and migration	SNP rs10474257 in RASA1 3’ UTR affects miR‐130a/miR‐19b binding, influencing angiogenesis and HCC risk.
miR‐let7g	rs3917	Controls cell growth, migration, invasion, and angiogenesis; regulates COL1A2.	COL1A2	ECM remodeling and metastasis	SNP rs3917 in COL1A2 disrupts let‐7g binding, increasing invasion and HCC susceptibility.
miR‐34a	rs1048638	Affects tumor progression, angiogenesis, and metastasis via NF‐*κ*B/HMGB1 axis.	CA9	Hypoxia response	SNP rs1048638 in CA9 3’ UTR impairs miR‐34a binding, enhancing HCC progression.
miR‐3976	rs6757	Regulates invasion, metastasis, and angiogenesis through CD147 interaction.	CD147	Tumor microenvironment	SNP rs6757 in CD147 3’ UTR affects miR‐3976 binding, influencing HCC risk.
miR‐367	rs1044129	Oncogenic role in HCC by activating PI3K/AKT pathway; regulates RYR3.	RYR3	Calcium signaling and tumor burden	SNP rs1044129 in RYR3 3’ UTR alters miR‐367 binding, impacting HCC survival.
miR‐300/miR‐381	rs1642742	miR‐300 upregulated in HCC; miR‐381 downregulated, affecting proliferation, migration, and invasion.	VHL	HIF signaling and tumor progression	SNP rs1642742 in VHL 3’ UTR enhances miR‐300/miR‐381 binding, contributing to HCC progression and metastasis.
miR‐502	rs16917496	Inhibits HCC cell proliferation, invasion, and metastasis.	SET8	Tumor growth inhibition	SNP rs16917496 affects miR‐502 binding to SET8, influencing HCC progression and survival.
miR‐431‐5p	rs112395617	Inhibits HCC cell proliferation and development.	JAK1	Tumor growth regulation	SNP rs112395617 disrupts miR‐431‐5p binding to JAK1, promoting HCC progression.
miR‐1269	rs73239138	Oncogene, regulates HCC cell proliferation.	SOX6	Tumor progression	SNP rs73239138 in miR‐1269 affects binding to SOX6, suppressing tumor growth in HCC.
miR‐151‐5p	rs56228771	Involved in tumor growth and metastasis.	SGSM3	Tumor progression and metastasis	SNP rs56228771 disrupts miR‐151‐5p binding to SGSM3, increasing HCC susceptibility.
MiR‐128‐3p	Expression negatively correlated with CYP2C9	Involved in cancer development, noninvasive biomarker for HCC survival	CYP2C9	Drug metabolism, detoxification	Alters CYP2C9 expression, affecting the metabolism of exogenous carcinogens and cancer drugs, with paradoxical effects on carcinogenesis in HCC.

**MicroRNA-130a** (miR‐130a) and **miR-19b** have been linked to the development of several diseases, including HCC [111]. MiR‐130a has been shown to dampen the NF‐*κ*B signaling pathway and its target gene *VEGFA* by reducing TNF‐*α* expression [112], contributing to accelerated invasion and migration in HCC [111]. Similarly, the miR‐19 family modulates angiogenic activity and the process of angiogenesis in endothelial cells, promoting proliferation or migration in various cancers [113, 114]. In a 2011 study by Ding et al., a screen of SNPs in the microRNA‐binding sites of 50 genes related to HCC identified six significant SNPs, including rs10474257 located within the miR‐19b and miR‐130a binding sites of *RASA1* [115]. *RASA1* encodes RAS P21 protein activator 1, whose levels are associated with liver cancer cells [116], and which regulates cell migration and angiogenesis. Defects in *RASA1* function can lead to irregular angiogenic remodeling of the capillary network, potentially contributing to HCC development [115].

**MicroRNA *let-7g*** plays crucial roles in regulating diverse biological processes [117] and is frequently dysregulated in various cancer types [117]. Research has demonstrated that let‐7 g influences cell growth, migration, and invasion when studied in HCC cell lines [115], as well as angiogenesis, vascular remodeling, and EMT [118]. Research by Zhu et al. demonstrated that the rs3917 polymorphism in the 3’UTR of *COL1A2* acts as a direct target site for *let-7* g [117]. *COL1A2* is responsible for encoding the alpha 2 chain of type I collagen, and its elevated expression impacts the invasion, migration, and growth of cancer cell lines [119]. Collagen I can promote the malignant properties of tumors by stimulating JNK1 signaling, which, through the upregulation of N‐cadherin levels, enhances invasion and metastasis [117]. Zhu et al.’s findings indicated that the rs3917 SNP in *COL1A2* could affect HCC risk, likely through modulation of *let-7g* binding and subsequent *COL1A2* expression [117].

**MicroRNA-34a** has been shown to influence almost all stages of cancer progression [120], including angiogenesis, cell migration, and tumorigenesis [120, 121]. Previous studies have highlighted the role of the miR‐34a/NF‐*κ*B/HMGB1 axis in angiogenesis in primary liver cancer [122], and upregulation of miR‐34a‐5p expression has been shown to decrease the invasive capacity of HCC cells [123]. A 2017 study by Hua et al. revealed that the rs1048638 polymorphism influences HCC risk and development by altering the expression of Carbonic Anhydrase IX (*CA9*) targeted by miR‐34a [107]. *CA9* prevents hypoxia‐induced upregulation of genes involved in angiogenesis, and knockdown of the DDX11‐AS1 axis, which is regulated by *CA9*, can significantly suppress cell proliferation, migration, and invasion [124]. Hua et al.’s research suggested that the *CA9* rs1048638 SNP disrupts the binding of miR‐34a to the *CA9* 3’UTR, thereby influencing HCC risk [107].

A **microRNA-3976** binding site SNP (rs6757) has been shown to significantly impact the differentiation, proliferation, metastasis, prognosis, and invasion of hepatocellular carcinoma by changing the quality of miR‐3976 binding to the *CD147* 3’UTR [108]. *CD147* encodes a glycoprotein that is significantly overexpressed on the surface of most malignant cancer cell types [125], and participates in tumor cell invasion, metastasis, and angiogenesis through various mechanisms. It is overexpressed in human cancers such as CNS, head and neck, breast, and gastrointestinal carcinomas [126, 127]. A study by Guo et al. demonstrated that the rs6757 SNP affects the efficiency of miR‐3976 binding to the *CD147* 3’UTR and affects HCC risk in the South Chinese population [108].

**MicroRNA-367** is influential in a variety of disease states, including cancer [128]. In HCC, miR‐367 acts as an oncogenic factor by activating the PI3K/AKT signaling pathway. Research has shown that when miR‐367 is inhibited, it can suppress cell proliferation, migration, and invasion. Peng et al. found that the rs1044129 polymorphism located in the 3’UTR of *RYR3* could alter the binding affinity between this SNP and miR‐367, which was linked to survival outcomes in HCC [109]. *RYR3* encodes the ryanodine receptor 3, a calcium channel that regulates cytosolic calcium concentrations [109]. Aberrant Ca2+ signaling is a hallmark of numerous cancers, and patients carrying *RYR* mutations have shown significantly higher tumor mutational burden (TMB) across most cancer types compared to those without these mutations [129]. Peng et al.’s work demonstrated that the rs1044129 SNP in the microRNA binding region of *RYR3* can serve as a potential biomarker for predicting outcomes in HCC [109].

**MicroRNA-300** and **miR-381** have been shown to regulate a variety of genes in cancer, and their expression levels often correlate with tumor stage and survival rates [130]. MiR‐300 is upregulated in HCC tissues [131], while miR‐381 is frequently downregulated in various cancers [132], and modulates multiple cellular functions including growth, cell cycle progression, migration, and invasion [133]. In a 2021 study by Chen et al., the rs1642742 polymorphism, located in the 3’UTR of the *VHL* gene, was shown to reduce *VHL* expression by enhancing its interaction with miR‐300 and miR‐381 [110]. The *VHL* gene produces the Von Hippel–Lindau tumor suppressor protein, and its mutations lead to the stabilization of HIF1*α* and HIF2*α*, which subsequently activate various oncogenic signaling pathways. Consequently, individuals with *VHL* mutations have an increased risk of developing various neoplasms [134]. Chen et al.’s study demonstrated that the *VHL* rs1642742 SNP plays a role in susceptibility to HCC and is associated with tumor growth and metastasis through its interaction with miR‐300 and miR‐381 [110].

## 6. MicroRNA Biogenesis and the Influence of SNPs

The process of miRNA biogenesis, which encompasses miRNA processing and maturation, exerts a significant influence on various aspects of cancer development [135, 136]. This multi‐step process is managed by a cohort of proteins collectively known as proteins involved in miRNA biogenesis [137]. Aberrant miRNA expression in malignancy is evidenced by its association with cancer‐related changes and by alterations in the expression or function of proteins crucial for miRNA production [138]. Prior research has demonstrated that SNPs within miRNA biogenesis genes, such as *RAN* and *DICER1*, can modulate the binding affinity of miRNAs to the 3’UTR of these genes, thereby affecting susceptibility to HCC (Table 2).

**MicroRNA-199-3p** (miR‐199‐3p) is involved in the initiation and progression of various cancers and modulates multiple signaling pathways [139]. The exportin 5 (XPO5)/RAN‐GTP complex is essential for the nuclear export of precursor miRNAs (pre‐miRNAs), and alterations in the expression or function of its components have been linked to cancer risk [136]. A 2013 study by Liu et al. indicated that the SNP rs3803012 could alter the binding capacity of miRNAs to the 3’UTR of the *RAN* gene [140]. The *RAN* gene encodes a small GTPase protein crucial for nuclear transport, including the translocation of pre‐miRNAs. Mutations in *RAN* can disrupt normal miRNA processing and expression, thereby contributing to the development and progression of tumor cells [141]. Specifically, the *RAN* rs3803012 variant was associated with the elevated risk of persistent HBV infection and subsequent HBV‐related HCC [140], suggesting a link between impaired pre‐miRNA transport and hepatocarcinogenesis.

**MicroRNA-574-3p** (miR‐574‐3p) has been identified as a crucial miRNA mediating various cellular processes [142]. Liu et al. also demonstrated that the *DICER1* SNP rs1057035 might alter the affinity of miR‐574‐3p to this gene and contribute to the risk of HBV‐related HCC [140]. *DICER1* encodes the DICER enzyme, a type III ribonuclease that plays a pivotal role in cleaving precursor miRNAs (pre‐miRNAs) into mature miRNAs. Consequently, DICER function is critical in the hepatocarcinogenesis process [141]. DICER is recognized as an essential component in the biogenesis of both miRNAs and small interfering RNAs (siRNAs) [143]. While DICER upregulation has been reported in several cancers [144], alterations in its activity due to genetic polymorphisms can disrupt the normal repertoire of mature miRNAs. In their study, Liu et al. hypothesized and provided evidence suggesting that the *DICER1* rs1057035 SNP is associated with an increased risk of HCC [140], potentially by affecting the levels or processing efficiency of specific miRNAs, including miR‐574‐3p.

## 7. MicroRNAs and the Impact of Single Nucleotide Polymorphisms on Tumor Growth and Progression

MicroRNAs exert significant influence on the intracellular signaling pathways activated by growth factors, which are critical regulators of tumor advancement, playing remarkable roles in this process and ultimately in tumor development [145]. Aberrations in the quantity and function of miRNAs are consistently associated with cancer initiation, progression, and metastasis [146]. MiRNAs can control not only tumor cells themselves but also the homeostasis of the tumor microenvironment, a critical factor underpinning tumor growth and dissemination [147]. During liver carcinogenesis, miRNAs are implicated in all facets of tumor cell behavior, including proliferation, survival, and migration [148]. Competitive endogenous RNAs (ceRNAs), which carry binding sites for microRNAs and compete with target mRNAs for miRNA binding, often underlie processes associated with cell differentiation and development [149]. Previous studies have shown that SNPs within miRNA binding sites of genes involved in tumor growth and progression, such as *ERBB4*, *EGFR*, *STAT3*, *ERBB2*, *JAK1*, *SOX6*, *SGSM3*, and *SET8*, can influence the risk and outcome of HCC [150–157] (Table 3).

**MicroRNA *let-7c*** is a compelling candidate for its involvement in the development of various diseases, particularly cancer [158]. It participates in organogenesis and fundamental cellular activities [159]. The *let-7* family of miRNAs generally functions as tumor suppressors in cancer cells, inhibiting metastasis and tumor growth [158]. Overexpression of *let-7c*, which can inhibit the Ras/NF*κ*B signaling pathway, has been shown to reduce malignant transformation and cancer stem cell development [158]. A study by Yu et al. indicated that the rs6147150 polymorphism is located within the seed region of miR‐*let-7c*, potentially targeting a sequence in the 3’UTR of *ERBB4* [150]. *ERBB4*, upon binding with epidermal growth factor (EGF), can activate downstream genes in the nucleus, thereby promoting cell division and proliferation [160]. According to Yu et al.’s research, the rs6147150 SNP, situated in the miR‐*let-7c* seed region, may contribute to HCC development in the Chinese population by affecting the post‐transcriptional regulation of *ERBB4* [150].

**MicroRNA-3196** (miR‐3196) is mainly involved in tumor development [161] and is significantly downregulated in HCC tissues, where its reduction promotes HCC cell growth [162]. In a study by Zhang et al., miR‐related SNPs in *EGFR* were investigated, revealing that the rs884225 polymorphism enhances the binding efficiency of miR‐3196 to the 3’UTR of *EGFR*, reducing epidermal growth factor receptor (EGFR) expression levels and suppressing cell proliferation [154]. The EGFR is a key component of a complex signaling pathway that regulates cancer cell growth, adhesion, metastasis, and survival [162]. MicroRNAs, including miR‐3196, play critical roles in modulating EGFR signaling [163]. EGFR can also facilitate EMT, promoting liver cancer progression [164]. Zhang et al.’s study suggested that rs884225 probably enhances miR‐3196 binding to *EGFR*, leading to decreased EGFR levels and consequently inhibiting cell proliferation in HCC [154].

**MicroRNA-214** (miR‐214) is linked to the development and proliferation of several tumors [165, 166]. This miRNA is located within the dynamin 3 gene at chromosome 19q13.3 [165] and has been found to exhibit tumor‐suppressing activity in HCC [167]. Fan et al. showed that miR‐214 might regulate the SNP rs111904020, leading to an increase in STAT3 levels [152]. Signal transducer and activator of transcription 3 (STAT3) is crucial in the pathogenesis of liver diseases [168]. STAT3 inhibition can promote apoptosis in cancer cells and prevent tumor growth [168], but it may also enhance the aggressiveness of HCC tumors by increasing the expression of EMT‐related proteins [169]. Fan et al.’s study indicated that the rs111904020 SNP in the *STAT3* 3’UTR acts as a factor that promotes HCC development by interfering with the regulatory role of miR‐214 on STAT3 expression [152].

**MicroRNA-221-5p** (miR‐221‐5p) has been extensively investigated for its role in cancer progression and its potential as a valuable biomarker in cancer research [170]. It acts as a regulator of chronic liver injury and inflammation‐associated processes in liver cells [170]. MiR‐221 may promote angiogenesis, and its overexpression can enhance cell growth and invasion [171, 172]. A study by Fan et al. proposed that miR‐221‐5p might regulate the rs113054794 SNP, leading to increased levels of ErbB2 in HCC patients [153]. Nuclear ErbB2 positivity has been strongly associated with histological indicators of hepatocellular injury [173]. ErbB2 has also been shown to inhibit the growth of HBx‐associated HCC cells [174]. Fan et al.’s study demonstrated that the rs113054794 SNP in the 3’UTR of *ERBB2* was associated with poor differentiation and larger tumor size in HCC by impeding the regulatory function of miR‐221‐5p on ErbB2 expression [153].

**MicroRNA-502** (miR‐502) expression levels have been associated with various HCC cell lines and patient liver tissues [175]. Overexpression of miR‐502 significantly suppresses HCC proliferation, tumor growth, invasion, and metastasis [175]. A study by Guo et al. showed that the polymorphism rs16917496 is located in the miR‐502 seed region within the 3’UTR of *SET8* in Chinese patients with hepatocellular carcinoma [151]. SET Domain Containing 8 (SET8) expression can be linked to the poor survival in HCC patients [176], and silencing of *SET8* can reduce the proliferation, migration, and invasion of tumor cells in HCC [177]. Guo et al.’s data indicate that *SET8* affects HCC outcomes by altering its expression, which depends on its binding affinity with miR‐502 [151].

**MicroRNA-431-5p** (miR‐431‐5p) can inhibit the development and progression of some cancers, including HCC [178]. Earlier research showed that it is notably reduced in HCC cells, and its upregulation restricted the growth of HCC cells [179]. A study by Yu et al. showed that the rs112395617 SNP in *JAK1* disrupted the binding site for miR‐431‐5p [155]. Mutations in *JAK1* have been observed in HCC [180]. Janus kinase 1 (JAK1) enhances growth factor and cytokine‐activated STAT signaling pathways and influences cellular growth, immune responses, and differentiation [155]. Yu et al. suggested that the rs112395617 polymorphism could play a role in HCC susceptibility by affecting *JAK1* transcriptional activity through interfering with its interaction with miR‐431‐5p [155].

**MicroRNA-1269** (miR‐1269) is crucial in controlling the progression of HCC [156]. Downregulation of miR‐1269 in liver cancer tissues has been associated with reduced proliferation of liver cancer cells [181]. MiR‐1269 holds diagnostic value in HCC and is also linked to a negative prognosis for the cancer [182]. Xiong et al. suggested that overexpression of miR‐1269 with a stronger binding ability to *SOX6* promoted cell proliferation in HCC [156]. SRY‐Box Transcription Factor 6 (SOX6) is expressed at low levels and acts as a promising prognostic biomarker for HCC [183], and its overexpression results in decreased cell growth and proliferation [184]. Results from Xiong et al. indicate that the SNP rs73239138 in the miR‐1269 binding site acts as a protective element that prevents binding to the 3’UTR of *SOX6*, therefore inhibiting tumor growth in patients with HCC [156].

The functional role of microRNA‐151‐5p (miR‐151‐5p) is influenced by the genetic characteristics of cancer tissue, with its expression levels ranging from downregulated to upregulated depending on the cancer type [185]. Expression of miR‐151‐5p is increased in liver cancer cells and is notably linked to their growth and spread [185]. A study showed that the rs56228771 SNP was located within the 3’UTR of *SGSM3*. Small G protein signaling modulator 3 (SGSM3) participates in the signaling pathway of small G protein‐coupled receptors, influencing susceptibility to hepatocellular carcinoma. Wang et al. indicated that rs56228771 interfered with a binding site for miR‐151‐5p, leading to an increase in SGSM3 levels, thus contributing to HCC risk [186].

## 8. MicroRNAs Effects on Drug Metabolism in HCC

**MicroRNA-128-3p** (miR‐128‐3p) is a remarkable factor in the development and pathogenesis of some cancers [187]. MiR‐128‐3p has potential as a noninvasive biomarker for predicting the overall survival of individuals with HCC [188]. Studies have shown an inverse correlation between the expression levels of miR‐128‐3p and Cytochrome P450 2C9 (CYP2C9) in HCC tumor tissues [189]. CYP2C9, a key enzyme predominantly expressed in the liver, plays a significant role in the metabolism of various therapeutic agents used in cancer treatment, as well as exogenous carcinogens [190]. Approximately 18% of the cytochrome P450 (CYP) proteins found in liver microsomes consist of CYP2C9 [191]. Alterations in the expression of *CYP* genes, such as *CYP2C9*, mediated by hsa‐miR‐128‐3p, can significantly influence the efficacy of xenobiotic detoxification processes. Furthermore, these changes can affect the generation of signaling molecules that modulate downstream signaling pathways, potentially exerting a paradoxical effect on HCC carcinogenesis (Table 3).

## 9. Conclusion

This review highlights the complex involvement of miRNAs and their associated SNPs in the pathogenesis, prognosis, and therapeutic response of HCC. The miRNA binding site polymorphisms are critical master modulators linking genetic variations to the dysregulation of key HCC pathogenic pathways (apoptosis, DNA repair, immune evasion, and metastasis). The interaction between miRNAs and SNPs within their binding sites increases the complexity of HCC. These genetic variations can modulate miRNA‐mRNA interactions, altering the expression of key genes and ultimately impacting HCC susceptibility, development, and treatment outcomes. The potential of miRNAs as noninvasive biomarkers could be utilized for timely diagnosis, prognosis, and monitoring therapeutic efficacy, which could improve patient management and outcomes. Future research, including large‐scale, multi‐ethnic cohort studies, functional validation using CRISPR‐Cas9 and in vivo models, and the development of SNP‐tailored combination therapies is crucial to fully understand the regulatory networks modulated by miRNAs and SNPs in HCC.

**Nomenclature**
HCC: Hepatocellular carcinoma
miRNAs: MicroRNAs
SNPs: Single nucleotide polymorphisms
ICC: Intrahepatic cholangiocarcinoma
HBV: Hepatitis B virus
HCV: Hepatitis C virus
IL‐6: Interleukin‐6
TNF‐*α*
: Tumor necrosis factor‐alpha
VEGF: Vascular endothelial growth factor
NAFLD: Nonalcoholic fatty liver disease
CT: Computed tomography
MRI: Magnetic resonance imaging
AFP: Alpha‐fetoprotein
AFP‐L3: Alpha‐fetoprotein‐L3
DCP: Des‐*γ*‐carboxy prothrombin
GPC3: Glypican‐3
GP73: Golgi protein‐73
cfDNA: Cell‐free DNA
CTCs: Circulating tumor cells
EVs: Extracellular vehicles
3’UTR: 3’ Untranslated region
BTRC: *β*‐Transducin repeat containing E3 ubiquitin protein ligase
SCF: Skp1‐Cullin‐F‐box
PLK1: Polo‐like kinase 1
DDB2: Damage‐specific DNA binding protein 2
NER: Nucleotide excision repair
RPA: Replication protein A
IFNAR: Interferon alpha/beta receptor
TLR4: Toll‐like receptor 4
PD‐1: Programmed cell death protein 1
IL‐1*α*
: Interleukin 1 alpha
RASA1: RAS P21 protein activator 1
CA9: Carbonic anhydrase IX
CNS: Central nervous system

## Consent

Not applicable.

## Conflicts of Interest

The authors declare no conflicts of interest.

## Author Contributions

S.A.R. mainly participated in literature search, study design, writing, and critical revision. F.K.M. and M.D. mainly participated in literature search, writing, and critical revision. S.M. and S.A. mainly participated in literature search, writing, critical revision, study design, and supervision. All authors read and approved the final manuscript.

## Funding

No funding was received for this manuscript.

## Data Availability

The data that support the findings of this study are available on request from the corresponding author.

## References

[1] Chidambaranathan-Reghupaty S. , Fisher P. B. , and Sarkar D. , Hepatocellular Carcinoma (HCC): Epidemiology, Etiology and Molecular Classification, Advances in Cancer Research. (2021) 149, 1–61, 10.1016/bs.acr.2020.10.001, 33579421. 33579421 PMC8796122

[2] McGlynn K. A. , Petrick J. L. , and El-Serag H. B. , Epidemiology of Hepatocellular Carcinoma, Hepatology. (2021) 73, no. S1, 4–13, 10.1002/hep.31288, 32319693. PMC7577946 32319693

[3] El-Serag H. B. and Rudolph K. L. , Hepatocellular Carcinoma: Epidemiology and Molecular Carcinogenesis, Gastroenterology. (2007) 132, no. 7, 2557–2576, 10.1053/j.gastro.2007.04.061, 2-s2.0-34250020201. 17570226

[4] Valderrama-Treviño A. I. , Barrera-Mera B. , Ceballos-Villalva J. C. , and Montalvo-Javé E. E. , Hepatic Metastasis From Colorectal Cancer, Euroasian Journal of Hepato-Gastroenterology. (2017) 7, no. 2, 166–175, 10.5005/jp-journals-10018-1241. 29201802 PMC5670263

[5] Ramakrishna G. , Rastogi A. , Trehanpati N. , Sen B. , Khosla R. , and Sarin S. K. , From Cirrhosis to Hepatocellular Carcinoma: New Molecular Insights on Inflammation and Cellular Senescence, Liver Cancer. (2013) 2, no. 3-4, 367–383, 10.1159/000343852, 24400224. 24400224 PMC3881319

[6] Llovet J. M. , Pinyol R. , Kelley R. K. , El-Khoueiry A. , Reeves H. L. , Wang X. W. , Gores G. J. , and Villanueva A. , Molecular Pathogenesis and Systemic Therapies for Hepatocellular Carcinoma, Nature Cancer. (2022) 3, no. 4, 386–401, 10.1038/s43018-022-00357-2. 35484418 PMC9060366

[7] Alqahtani A. , Khan Z. , Alloghbi A. , Said Ahmed T. S. , Ashraf M. , and Hammouda D. M. , Hepatocellular Carcinoma: Molecular Mechanisms and Targeted Therapies, Medicina. (2019) 55, no. 9, 10.3390/medicina55090526, 2-s2.0-85071766181, 31450841. PMC6780754 31450841

[8] Cha C. and Dematteo R. P. , Molecular Mechanisms in Hepatocellular Carcinoma Development, Best Practice & Research Clinical Gastroenterology. (2005) 19, no. 1, 25–37, 10.1016/j.bpg.2004.11.005, 2-s2.0-14744291990. 15757803

[9] Luo X. , He X. , Zhang X. , Zhao X. , Zhang Y. , Shi Y. , and Hua S. , Hepatocellular Carcinoma: Signaling Pathways, Targeted Therapy, and Immunotherapy, MedComm. (2024) 5, no. 2, 10.1002/mco2.474, 38318160. PMC10838672 38318160

[10] Qi Y. , Song Y. , Cai M. , Li J. , Yu Z. , Li Y. , Huang J. , Jiang Y. , Peng C. , Jiang B. , and Liu S. , Vascular Endothelial Growth Factor a Is a Potential Prognostic Biomarker and Correlates With Immune Cell Infiltration in Hepatocellular Carcinoma, Journal of Cellular and Molecular Medicine. (2023) 27, no. 4, 538–552, 10.1111/jcmm.17678, 36729917. 36729917 PMC9930434

[11] Polyzos S. A. , Chrysavgis L. , Vachliotis I. D. , Chartampilas E. , and Cholongitas E. , Nonalcoholic Fatty Liver Disease and Hepatocellular Carcinoma: Insights in Epidemiology, Pathogenesis, Imaging, Prevention and Therapy, Seminars in Cancer Biology. (2023) 93, 20–35, 10.1016/j.semcancer.2023.04.010, 37149203. 37149203

[12] Nguyen M. H. , Roberts L. R. , Engel-Nitz N. M. , Bancroft T. , Ozbay A. B. , and Singal A. G. , Gaps in Hepatocellular Carcinoma Surveillance in a United States Cohort of Insured Patients With Cirrhosis, Current Medical Research and Opinion. (2022) 38, no. 12, 2163–2173, 10.1080/03007995.2022.2124070, 36111416. 36111416

[13] Parikh N. D. , Mehta A. S. , Singal A. G. , Block T. , Marrero J. A. , and Lok A. S. , Biomarkers for the Early Detection of Hepatocellular Carcinoma, Cancer Epidemiology, Biomarkers & Prevention. (2020) 29, no. 12, 2495–2503, 10.1158/1055-9965.Epi-20-0005. PMC7529652 32238405

[14] Zhou J. , Sun H. , Wang Z. , Cong W. , Wang J. , Zeng M. , Zhou W. , Bie P. , Liu L. , Wen T. , Han G. , Wang M. , Liu R. , Lu L. , Ren Z. , Chen M. , Zeng Z. , Liang P. , Liang C. , Chen M. , Yan F. , Wang W. , Ji Y. , Yun J. , Cai D. , Chen Y. , Cheng W. , Cheng S. , Dai C. , Guo W. , Hua B. , Huang X. , Jia W. , Li Y. , Li Y. , Liang J. , Liu T. , Lv G. , Mao Y. , Peng T. , Ren W. , Shi H. , Shi G. , Tao K. , Wang W. , Wang X. , Wang Z. , Xiang B. , Xing B. , Xu J. , Yang J. , Yang J. , Yang Y. , Yang Y. , Ye S. , Yin Z. , Zhang B. , Zhang B. , Zhang L. , Zhang S. , Zhang T. , Zhao Y. , Zheng H. , Zhu J. , Zhu K. , Liu R. , Shi Y. , Xiao Y. , Dai Z. , Teng G. , Cai J. , Wang W. , Cai X. , Li Q. , Shen F. , Qin S. , Dong J. , and Fan J. , Guidelines for the Diagnosis and Treatment of Hepatocellular Carcinoma (2019 Edition), Liver Cancer. (2020) 9, no. 6, 682–720, 10.1159/000509424, 33442540. 33442540 PMC7768108

[15] McMahon B. , Cohen C. , Brown R. S.Jr., El-Serag H. , Ioannou G. N. , Lok A. S. , Roberts L. R. , Singal A. G. , and Block T. , Opportunities to Address Gaps in Early Detection and Improve Outcomes of Liver Cancer, JNCI Cancer Spectr. (2023) 7, no. 3, 10.1093/jncics/pkad034. PMC10212536 37144952

[16] Hong S. B. , Kim D. H. , Choi S. H. , Kim S. Y. , Lee J. S. , Lee N. K. , and Choi J. I. , Inadequate Ultrasound Examination in Hepatocellular Carcinoma Surveillance: A Systematic Review and Meta-Analysis, Journal of Clinical Medicine. (2021) 10, no. 16, 10.3390/jcm10163535, 34441831. PMC8397222 34441831

[17] Hanif H. , Ali M. J. , Susheela A. T. , Khan I. W. , Luna-Cuadros M. A. , Khan M. M. , and Lau D. T. , Update on the Applications and Limitations of Alpha-Fetoprotein for Hepatocellular Carcinoma, World Journal of Gastroenterology. (2022) 28, no. 2, 216–229, 10.3748/wjg.v28.i2.216, 35110946. 35110946 PMC8776528

[18] Marrero J. A. , Feng Z. , Wang Y. , Nguyen M. H. , Befeler A. S. , Roberts L. R. , Reddy K. R. , Harnois D. , Llovet J. M. , Normolle D. , Dalhgren J. , Chia D. , Lok A. S. , Wagner P. D. , Srivastava S. , and Schwartz M. , *α*-Fetoprotein, Des-*γ* Carboxyprothrombin, and Lectin-Bound *α*-Fetoprotein in Early Hepatocellular Carcinoma, Gastroenterology. (2009) 137, no. 1, 110–118, 10.1053/j.gastro.2009.04.005, 2-s2.0-67649305155, 19362088. 19362088 PMC2704256

[19] Khare S. , Khare T. , Ramanathan R. , and Ibdah J. A. , Hepatocellular Carcinoma: The Role of MicroRNAs, Biomolecules. (2022) 12, no. 5, 10.3390/biom12050645. PMC9138333 35625573

[20] von Felden J. , Garcia-Lezana T. , Schulze K. , Losic B. , and Villanueva A. , Liquid Biopsy in the Clinical Management of Hepatocellular Carcinoma, Gut. (2020) 69, no. 11, 2025–2034, 10.1136/gutjnl-2019-320282. 32883873

[21] Jeepalyam S. , Sheel A. , Ejaz A. , Miller E. , and Manne A. , Is Cell-Free DNA Testing in Hepatocellular Carcinoma Ready for Prime Time?, International Journal of Molecular Sciences. (2023) 24, no. 18, 14231, 10.3390/ijms241814231, 37762533. 37762533 PMC10531802

[22] Juratli M. A. , Pollmann N. S. , Oppermann E. , Mohr A. , Roy D. , Schnitzbauer A. , Michalik S. , Vogl T. , Stoecklein N. H. , Houben P. , Katou S. , Becker F. , Hoelzen J. P. , Andreou A. , Pascher A. , Bechstein W. O. , and Struecker B. , Extracellular Vesicles as Potential Biomarkers for Diagnosis and Recurrence Detection of Hepatocellular Carcinoma, Scientific Reports. (2024) 14, no. 1, 10.1038/s41598-024-55888-8, 38438456. PMC10912302 38438456

[23] Chen L. , Wu T. , Fan R. , Qian Y. S. , Liu J. F. , Bai J. , Zheng B. , Liu X. L. , Zheng D. , Du L. T. , Jiang G. Q. , Wang Y. C. , Fan X. T. , Deng G. H. , Wang C. Y. , Shen F. , Hu H. P. , Zhang Q. Z. , Ye Y. N. , Zhang J. , Gao Y. H. , Xia J. , Yan H. D. , Liang M. F. , Yu Y. L. , Sun F. M. , Gao Y. J. , Sun J. , Zhong C. X. , Wang Y. , Wang H. , Kong F. , Chen J. M. , Wen H. , Wu B. M. , Wang C. X. , Wu L. , Hou J. L. , and Wang H. Y. , Cell-Free DNA Testing for Early Hepatocellular Carcinoma Surveillance, eBioMedicine. (2024) 100, 104962, 10.1016/j.ebiom.2023.104962, 38184937. 38184937 PMC10808903

[24] Johnson P. , Zhou Q. , Dao D. Y. , and Lo Y. D. , Circulating Biomarkers in the Diagnosis and Management of Hepatocellular Carcinoma, Nature Reviews Gastroenterology & Hepatology. (2022) 19, no. 10, 670–681, 10.1038/s41575-022-00620-y. 35676420

[25] Lv Y. and Sun X. , Role of miRNA in Pathogenesis, Diagnosis, and Prognosis in Hepatocellular Carcinoma, Chemical Biology & Drug Design. (2024) 103, no. 1, e14352, 10.1111/cbdd.14352. 37726253

[26] Beilankouhi E. A. V. , Maghsoodi M. S. , Sani M. Z. , Khosroshahi N. S. , Zarezadeh R. , Nargesi M. M. , Safaralizadeh R. , and Valilo M. , miRNAs That Regulate Apoptosis in Breast Cancer and Cervical Cancer, Cell Biochemistry and Biophysics. (2024) 82, no. 3, 1993–2006, 10.1007/s12013-024-01405-7. 38969951

[27] Braconi C. , Henry J. C. , Kogure T. , Schmittgen T. , and Patel T. , The Role of MicroRNAs in Human Liver Cancers, Proceedings of the Seminars in oncology, 2011, Elsevier, 752–763. 10.1053/j.seminoncol.2011.08.001 PMC3928803 22082761

[28] Li M.-P. , Hu Y.-D. , Hu X.-L. , Zhang Y.-J. , Yang Y.-L. , Jiang C. , Tang J. , and Chen X.-P. , MiRNAs and miRNA Polymorphisms Modify Drug Response, International Journal of Environmental Research and Public Health. (2016) 13, no. 11, 10.3390/ijerph13111096, 2-s2.0-84995404169, 27834829. PMC5129306 27834829

[29] Xu X. , Tao Y. , Shan L. , Chen R. , Jiang H. , Qian Z. , Cai F. , Ma L. , and Yu Y. , The Role of MicroRNAs in Hepatocellular Carcinoma, Journal of Cancer. (2018) 9, no. 19, 3557–3569, 10.7150/jca.26350, 2-s2.0-85053031778. 30310513 PMC6171016

[30] Ma R. , Zhao M. , Zou X. , Zhou J. , and Bai Z. , MicroRNA Polymorphism: A Target for Diagnosis and Prognosis of Hepatocellular Carcinoma?, Oncology Letters. (2021) 21, no. 4, 1–7, 10.3892/ol.2021.12586. 33692856 PMC7933756

[31] Zheng B. H. , Ni X. J. , and Liu H. B. , Exosomal MicroRNAs in Hepatocellular Carcinoma, Expanding Research Field, World Journal of Gastroenterology. (2024) 30, no. 20, 2618–2620, 10.3748/wjg.v30.i20.2618, 38855155. 38855155 PMC11154679

[32] El-Huneidi W. , Eladl M. A. , and Muhammad J. S. , Single Nucleotide Polymorphisms in microRNA Binding Sites on the HOX Genes Regulate Carcinogenesis: An In-Silico Approach, Biochemistry and Biophysics Reports. (2021) 27, 101083, 10.1016/j.bbrep.2021.101083, 34368470. 34368470 PMC8326182

[33] Landi D. , Gemignani F. , and Landi S. , Role of Variations Within MicroRNA-Binding Sites in Cancer, Mutagenesis. (2012) 27, no. 2, 205–210, 10.1093/mutage/ger055, 2-s2.0-84856940711, 22294768. 22294768

[34] Lynam-Lennon N. , Maher S. G. , and Reynolds J. V. , The Roles of MicroRNA in Cancer and Apoptosis, Biological Reviews of the Cambridge Philosophical Society. (2009) 84, no. 1, 55–71, 10.1111/j.1469-185X.2008.00061.x, 2-s2.0-58849139162. 19046400

[35] Xu J. , Wang Y. , Tan X. , and Jing H. , MicroRNAs in Autophagy and Their Emerging Roles in Crosstalk With Apoptosis, Autophagy. (2012) 8, no. 6, 873–882, 10.4161/auto.19629, 2-s2.0-84864872512, 22441107. 22441107 PMC3427253

[36] Elsarraj H. S. , Stecklein S. R. , Valdez K. , and Behbod F. , Emerging Functions of MicroRNA-146a/b in Development and Breast Cancer: MicroRNA-146a/b in Development and Breast Cancer, Journal of Mammary Gland Biology and Neoplasia. (2012) 17, no. 1, 79–87, 10.1007/s10911-012-9240-x, 2-s2.0-84858703919, 22350993. 22350993 PMC8276881

[37] Nahand J. S. , Karimzadeh M. R. , Nezamnia M. , Fatemipour M. , Khatami A. , Jamshidi S. , Moghoofei M. , Taghizadieh M. , Hajighadimi S. , Shafiee A. , Sadeghian M. , Bokharaei-Salim F. , and Mirzaei H. , The Role of miR-146a in Viral Infection, IUBMB Life. (2020) 72, no. 3, 343–360, 10.1002/iub.2222. 31889417

[38] Mahdavi F. S. , Mardi S. , Mohammadi S. , Ansari S. , Yaslianifard S. , Fallah P. , and Mozhgani S.-H. , MicroRNA-146: Biomarker and Mediator of Cardiovascular Disease, Disease Markers. (2022) 2022, no. 1, 7767598, 10.1155/2022/7767598. 39281713 PMC11401689

[39] Ren Y. , Wang X. , Ji T. , and Cai X. , MicroRNA-146b-5p Suppresses Cholangiocarcinoma Cells by Targeting TRAF6 and Modulating p53 Translocation, Acta Histochemica. (2021) 123, no. 7, 151793, 10.1016/j.acthis.2021.151793, 34610483. 34610483

[40] Kadioglu D. B. , Demirtas C. O. , Pirim D. , Dilber F. , and Eren F. , The Preliminary Data of Gene Expressions and Bioinformatics Analysis of miR-146b-5p and miR-4510 in the Turkish Population in HBV-Related Hepatocellular Carcinoma, Hepatology Forum. (2024) 5, no. 3, 106–112, 10.14744/hf.2023.2023.0054, 39006138. 39006138 PMC11237244

[41] Wojtas B. , Ferraz C. , Stokowy T. , Hauptmann S. , Lange D. , Dralle H. , Musholt T. , Jarzab B. , Paschke R. , and Eszlinger M. , Differential miRNA Expression Defines Migration and Reduced Apoptosis in Follicular Thyroid Carcinomas, Molecular and Cellular Endocrinology. (2014) 388, no. 1-2, 1–9, 10.1016/j.mce.2014.02.011, 2-s2.0-84896455861, 24631480. 24631480

[42] Zhong X. , Huang S. , Liu D. , Jiang Z. , Jin Q. , Li C. , Da L. , Yao Q. , and Wang D. , Galangin Promotes Cell Apoptosis Through Suppression of H19 Expression in Hepatocellular Carcinoma Cells, Cancer Medicine. (2020) 9, no. 15, 5546–5557, 10.1002/cam4.3195, 32485786. 32485786 PMC7402821

[43] Gan L. , Liao S. , Tong Y. , Li W. , Peng W. , and Deng S. , Long Noncoding RNA H19 Mediates Neural Stem/Progenitor Cells Proliferation, Differentiation and Apoptosis Through the p53 Signaling Pathway After Ischemic Stroke, Biochemical and Biophysical Research Communications. (2022) 597, 8–15, 10.1016/j.bbrc.2022.01.095, 35121179. 35121179

[44] Song Y. , Xing H. , Zhou L. , Zhang N. , and Yang M. , LncRNA H19 Modulated by miR-146b-3p/miR-1539-Mediated Allelic Regulation in Transarterial Chemoembolization of Hepatocellular Carcinoma, Archives of Toxicology. (2021) 95, no. 9, 3063–3070, 10.1007/s00204-021-03119-8, 34251499. 34251499

[45] Korać P. , Antica M. , and Matulić M. , MiR-7 in Cancer Development, Biomedicine. (2021) 9, no. 3. 10.3390/biomedicines9030325 PMC8004586 33806891

[46] Zhao J. , Zhou Y. , Guo M. , Yue D. , Chen C. , Liang G. , and Xu L. , MicroRNA-7: Expression and Function in Brain Physiological and Pathological Processes, Cell & Bioscience. (2020) 10, no. 1, 10.1186/s13578-020-00436-w, 32537124. PMC7288475 32537124

[47] Fang D.-Z. , Wang Y.-P. , Liu J. , Hui X.-B. , Wang X.-D. , Chen X. , and Liu D. , MicroRNA-129-3p Suppresses Tumor Growth by Targeting E2F5 in Glioblastoma, European Review for Medical & Pharmacological Sciences. (2018) 22, no. 4. 10.26355/eurrev_201802_14387 29509253

[48] Shao W. , Azam Z. , Guo J. , and To S. S. T. , Oncogenic Potential of PIK3CD in Glioblastoma Is Exerted Through Cytoskeletal Proteins PAK3 and PLEK2, Laboratory Investigation. (2022) 102, no. 12, 1314–1322, 10.1038/s41374-022-00821-8, 35851857. 35851857

[49] Yazarlou F. , Kadkhoda S. , and Ghafouri-Fard S. , Emerging Role of Let-7 Family in the Pathogenesis of Hematological Malignancies, Biomedicine & Pharmacotherapy. (2021) 144, 112334, 10.1016/j.biopha.2021.112334. 34656064

[50] Pessôa R. L. , da Rosa A. G. , and de Oliveira R. B. , MicroRNA Let-7 Plays an Important Role in the Immunopathology of COVID-19: A Systematic Review, Immuno. (2023) 3, no. 1, 112–121, 10.3390/immuno3010008.

[51] Messina S. , The RAS Oncogene in Brain Tumors and the Involvement of Let-7 microRNA, Molecular Biology Reports. (2024) 51, no. 1, 10.1007/s11033-024-09439-z, 38637419. PMC11026240 38637419

[52] Ma J. , Guo R. , Wang T. , Pan X. , and Lei X. , Let-7b Binding Site Polymorphism in the B-Cell Lymphoma-Extra Large 3′UTR Is Associated With Fluorouracil Resistance of Hepatocellular Carcinoma, Molecular Medicine Reports. (2015) 11, no. 1, 677–681, 10.3892/mmr.2014.2692, 2-s2.0-84916216223, 25333670. 25333670

[53] Morales-Martínez M. and Vega M. I. , Roles and Regulation of BCL-xL in Hematological Malignancies, International Journal of Molecular Sciences. (2022) 23, no. 4, 10.3390/ijms23042193, 35216310. PMC8876520 35216310

[54] Czabotar P. E. , Lessene G. , Strasser A. , and Adams J. M. , Control of Apoptosis by the BCL-2 Protein Family: Implications for Physiology and Therapy, Nature Reviews Molecular Cell Biology. (2014) 15, no. 1, 49–63, 10.1038/nrm3722, 2-s2.0-84890909335, 24355989. 24355989

[55] Wang Y. , Chen R. , Zhou X. , Guo R. , Yin J. , Li Y. , and Ma G. , miR-137: A Novel Therapeutic Target for Human Glioma, Molecular Therapy-Nucleic Acids. (2020) 21, 614–622, 10.1016/j.omtn.2020.06.028, 32736290. 32736290 PMC7393316

[56] Liu S. , Ruan Y. , Chen X. , He B. , and Chen Q. , miR-137: A Potential Therapeutic Target for Lung Cancer, Frontiers in Cell and Developmental Biology. (2024) 12, 1427724, 10.3389/fcell.2024.1427724, 39247624. 39247624 PMC11377224

[57] Tan C. , Liu S. , Tan S. , Zeng X. , Yu H. , Li A. , Bei C. , and Qiu X. , Polymorphisms in microRNA Target Sites of Forkhead Box O Genes Are Associated With Hepatocellular Carcinoma, PLoS One. (2015) 10, no. 3, e0119210, 10.1371/journal.pone.0119210, 2-s2.0-84928905842. 25739100 PMC4357486

[58] Remadevi V. , Muraleedharan P. , and Sreeja S. , FOXO1: A Pivotal Pioneer Factor in Oral Squamous Cell Carcinoma, American Journal of Cancer Research. (2021) 11, no. 10, 4700–4710, 34765288. 34765288 PMC8569351

[59] Zhang X. , Tang N. , Hadden T. J. , and Rishi A. K. , Akt, FoxO and regulation of Apoptosis, Biochimica et Biophysica Acta (BBA)-Molecular Cell Research. (2011) 1813, no. 11, 1978–1986, 10.1016/j.bbamcr.2011.03.010, 2-s2.0-80052962211, 21440011. 21440011

[60] Jin M. H. and Oh D.-Y. , ATM in DNA Repair in Cancer, Pharmacology & Therapeutics. (2019) 203, 107391, 10.1016/j.pharmthera.2019.07.002, 2-s2.0-85068828999. 31299316

[61] Li Y. , Tong Y. , Liu J. , and Lou J. , The Role of MicroRNA in DNA Damage Response, Frontiers in Genetics. (2022) 13, 850038, 10.3389/fgene.2022.850038. 35591858 PMC9110863

[62] Zhu L. , Sturgis E. M. , Lu Z. , Zhang H. , Wei P. , Wei Q. , and Li G. , Association Between miRNA-Binding Site Polymorphisms in Double-Strand Break Repair Genes and Risk of Recurrence in Patients With Squamous Cell Carcinomas of the Non-Oropharynx, Carcinogenesis. (2017) 38, no. 4, 432–438, 10.1093/carcin/bgx019, 2-s2.0-85019642375. 28334093 PMC6075523

[63] Li Z. , Guo Y. , Zhou L. , Ge Y. , Wei L. , Li L. , Zhou C. , Wei J. , Yuan Q. , Li J. , and Yang M. , Association of a Functional RAD52 Genetic Variant Locating in a miRNA Binding Site With Risk of HBV-Related Hepatocellular Carcinoma, Molecular Carcinogenesis. (2015) 54, no. 9, 853–858, 10.1002/mc.22156, 2-s2.0-84938970942, 24729511. 24729511

[64] Chen S. , He Y. , Ding J. , Jiang Y. , Jia S. , Xia W. , Zhao J. , Lu M. , Gu Z. , and Gao Y. , An Insertion/Deletion Polymorphism in the 3′ Untranslated Region of Beta-Transducin Repeat-Containing Protein (betaTrCP) Is Associated With Susceptibility for Hepatocellular Carcinoma in Chinese, Biochemical and Biophysical Research Communications. (2010) 391, no. 1, 552–556, 10.1016/j.bbrc.2009.11.096, 2-s2.0-72949115258, 19931512. 19931512

[65] Qiu M. , Liu Y. , Zhou Z. , Jiang Y. , Lin Q. , Huo R. , Liang X. , Yu X. , Zhou X. , and Yu H. , Association between Single-Nucleotide Polymorphism in MicroRNA Target Site of *DDB2* and Risk of Hepatocellular Carcinoma in a Southern Chinese Population, BioMed Research International. (2020) 2020, no. 1, 10.1155/2020/8528747, 32090112, 8528747. 32090112 PMC7031712

[66] Qiu M. , Liu Y. , Lin Q. , Jiang Y. , Zhou Z. , Wen Q. , Liang X. , Zhou X. , and Yu H. , A Functional Variant in the RAD51 3′ UTR Is Associated With Survival of Hepatocellular Carcinoma Patients, Gene. (2023) 851, 146964, 10.1016/j.gene.2022.146964, 36261080. 36261080

[67] Xie J. , He C. , Su Y. , Ding Y. , Zhu X. , Xu Y. , Ding J. , Zhou H. , and Wang H. , Research Progress on MicroRNA in Gout, Frontiers in Pharmacology. (2022) 13, 981799, 10.3389/fphar.2022.981799, 36339582. 36339582 PMC9631428

[68] Saitoh T. and Katoh M. , Expression Profiles of *β*TRCP1 and *β*TRCP2, and Mutation Analysis of *β*TRCP2 in Gastric Cancer, International Journal of Oncology. (2001) 18, no. 5, 959–964, 10.3892/ijo.18.5.959, 11295041. 11295041

[69] Giráldez S. , Galindo-Moreno M. , Limón-Mortés M. C. , Rivas A. C. , Herrero-Ruiz J. , Mora-Santos M. , Sáez C. , Japón M. , Tortolero M. , and Romero F. , G(1)/S Phase Progression Is Regulated by PLK1 Degradation Through the CDK1/*β*TrCP Axis, FASEB Journal. (2017) 31, no. 7, 2925–2936, 10.1096/fj.201601108R, 2-s2.0-85021693447, 28360195. 28360195

[70] Wang D. , Chen X. , Yu D. D. , Yang S. J. , Shen H. Y. , Sha H. , Zhong S. L. , Zhao J. H. , and Tang J. H. , miR-197: A Novel Biomarker for Cancers, Gene. (2016) 591, no. 2, 313–319, 10.1016/j.gene.2016.06.035, 2-s2.0-84977546936, 27320730. 27320730

[71] Andrade A. C. , Freitas T. R. , Dornelas G. G. , Gomes L. C. , Barbosa B. L. , Araújo S. S. , Gomes K. B. , and Sabino A. P. , miR-197, miR-26a And miR-27a Analysis in Chronic Lymphocytic Leukemia, Biomarkers in Medicine. (2022) 16, no. 12, 903–914, 10.2217/bmm-2021-0873. 35833845

[72] Luo J. , Zhou J. , Cheng Q. , Zhou C. , and Ding Z. , Role of MicroRNA-133a in Epithelial Ovarian Cancer Pathogenesis and Progression, Oncology Letters. (2014) 7, no. 4, 1043–1048, 10.3892/ol.2014.1841, 2-s2.0-84896735110, 24944666. 24944666 PMC3961467

[73] Dong Y. , Zhao J. , Wu C. W. , Zhang L. , Liu X. , Kang W. , Leung W. W. , Zhang N. , Chan F. K. , Sung J. J. , Ng S. S. , and Yu J. , Tumor Suppressor Functions of miR-133a in Colorectal Cancer, Molecular Cancer Research. (2013) 11, no. 9, 1051–1060, 10.1158/1541-7786.MCR-13-0061, 2-s2.0-84884481056, 23723074. 23723074

[74] Gilson P. , Drouot G. , Witz A. , Merlin J. L. , Becuwe P. , and Harlé A. , Emerging Roles of DDB2 in Cancer, International Journal of Molecular Sciences. (2019) 20, no. 20, 10.3390/ijms20205168, 2-s2.0-85073656614, 31635251. PMC6834144 31635251

[75] Deng B. , Tang X. , and Wang Y. , Role of microRNA-129 in Cancer and Non-Cancerous Diseases (Review), Experimental and Therapeutic Medicine. (2021) 22, no. 3, 10.3892/etm.2021.10350, 34335879. PMC8290460 34335879

[76] Gao Y. , Xu L. , Li Y. , Qi D. , Wang C. , Luan C. , Zheng S. , Du Q. , Liu W. , and Lu G. , Calcium Transferring From ER to Mitochondria via miR-129/ITPR2 Axis Controls Cellular Senescence In Vitro and In Vivo, Mechanisms of Ageing and Development. (2024) 218, 111902, 10.1016/j.mad.2024.111902. 38218462

[77] Angelis K. J. , Záveská Drábková L. , Vágnerová R. , and Holá M. , RAD51 and RAD51B Play Diverse Roles in the Repair of DNA Double Strand Breaks in Physcomitrium Patens, Genes. (2023) 14, no. 2, 10.3390/genes14020305. PMC9956106 36833232

[78] Letafati A. , Najafi S. , Mottahedi M. , Karimzadeh M. , Shahini A. , Garousi S. , Abbasi-Kolli M. , Sadri Nahand J. , Tamehri Zadeh S. S. , Hamblin M. R. , Rahimian N. , Taghizadieh M. , and Mirzaei H. , MicroRNA Let-7 and Viral Infections: Focus on Mechanisms of Action, Cellular & Molecular Biology Letters. (2022) 27, no. 1, 10.1186/s11658-022-00317-9, 35164678. PMC8853298 35164678

[79] Xue C. and Greene E. C. , New Roles for RAD52 in DNA Repair, Cell Research. (2018) 28, no. 12, 1127–1128, 10.1038/s41422-018-0105-8, 2-s2.0-85055707008. 30367126 PMC6274643

[80] Disis M. L. , Immune Regulation of Cancer, Journal of Clinical Oncology. (2010) 28, no. 29, 4531–4538, 10.1200/JCO.2009.27.2146, 2-s2.0-78149432308. 20516428 PMC3041789

[81] Jia Y. and Wei Y. , Modulators of MicroRNA Function in the Immune System, International Journal of Molecular Sciences. (2020) 21, no. 7, 10.3390/ijms21072357. PMC7177468 32235299

[82] Yi M. , Xu L. , Jiao Y. , Luo S. , Li A. , and Wu K. , The Role of Cancer-Derived microRNAs in Cancer Immune Escape, Journal of Hematology & Oncology. (2020) 13, no. 1, 10.1186/s13045-020-00848-8, 32222150. PMC7103070 32222150

[83] Karimzadeh M. R. , Zarin M. , Ehtesham N. , Khosravi S. , Soosanabadi M. , Mosallaei M. , and Pourdavoud P. , MicroRNA Binding Site Polymorphism in Inflammatory Genes Associated With Colorectal Cancer: Literature Review and Bioinformatics Analysis, Cancer Gene Therapy. (2020) 27, no. 10-11, 739–753, 10.1038/s41417-020-0172-0, 32203060. 32203060

[84] Jiang Z.-C. , Tang X.-M. , Zhao Y.-R. , and Zheng L. , A Functional Variant at miR-34a Binding Site in Toll-Like Receptor 4 Gene Alters Susceptibility to Hepatocellular Carcinoma in a Chinese Han Population, Tumor Biology. (2014) 35, no. 12, 12345–12352, 10.1007/s13277-014-2547-z, 2-s2.0-84925285265, 25179842. 25179842

[85] Gao Y. , He Y. , Ding J. , Wu K. , Hu B. , Liu Y. , Wu Y. , Guo B. , Shen Y. , Landi D. , Landi S. , Zhou Y. , and Liu H. , An Insertion/Deletion Polymorphism at miRNA-122-Binding Site in the Interleukin-1alpha 3′ Untranslated Region Confers Risk for Hepatocellular Carcinoma, Carcinogenesis. (2009) 30, no. 12, 2064–2069, 10.1093/carcin/bgp283, 2-s2.0-73949086088, 19917630. 19917630

[86] Zhou C. , Yu Q. , Chen L. , Wang J. , Zheng S. , and Zhang J. , A miR-1231 Binding Site Polymorphism in the 3’UTR of IFNAR1 Is Associated With Hepatocellular Carcinoma Susceptibility, Gene. (2012) 507, no. 1, 95–98, 10.1016/j.gene.2012.06.073, 2-s2.0-84864974809. 22824466

[87] Zhang G. , Li N. , Li Z. , Zhu Q. , Li F. , Yang C. , Han Q. , Lv Y. , Zhou Z. , and Liu Z. , microRNA-4717 Differentially Interacts With its Polymorphic Target in the PD1 3’ untranslated Region: A Mechanism for Regulating PD-1 Expression and Function in HBV-Associated Liver Diseases, Oncotarget. (2015) 6, no. 22, 18933–18944, 10.18632/oncotarget.3662, 2-s2.0-84938894232, 25895129. 25895129 PMC4662465

[88] Wang Y. , Zhang Q. , Guo B. , Feng J. , and Zhao D. , miR-1231 Is Downregulated in Prostate Cancer With Prognostic and Functional Implications, Oncology Research and Treatment. (2020) 43, no. 3, 78–86, 10.1159/000504606, 31822000. 31822000

[89] Zhang J. , Zhang J. , Qiu W. , Zhang J. , Li Y. , Kong E. , Lu A. , Xu J. , and Lu X. , MicroRNA-1231 Exerts a Tumor Suppressor Role Through Regulating the EGFR/PI3K/AKT Axis in Glioma, Journal of Neuro-Oncology. (2018) 139, no. 3, 547–562, 10.1007/s11060-018-2903-8, 2-s2.0-85047139819, 29774498. 29774498 PMC6132976

[90] Müller L. , Aigner P. , and Stoiber D. , Type I Interferons and Natural Killer Cell Regulation in Cancer, Frontiers in Immunology. (2017) 8, 10.3389/fimmu.2017.00304, 2-s2.0-85017110803, 28408907. PMC5374157 28408907

[91] López-Bielma M. F. , Falfán-Valencia R. , Abarca-Rojano E. , and Pérez-Rubio G. , Participation of Single-Nucleotide Variants in IFNAR1 and IFNAR2 in the Immune Response Against SARS-CoV-2 Infection: A Systematic Review, Pathogens. (2023) 12, no. 11, 10.3390/pathogens12111320. PMC10675296 38003785

[92] Taheri F. , Ebrahimi S. O. , Shareef S. , and Reiisi S. , Regulatory and Immunomodulatory Role of miR-34a in T Cell Immunity, Life Sciences. (2020) 262, 118209, 10.1016/j.lfs.2020.118209, 32763292. 32763292

[93] Shetab Boushehri M. A. and Lamprecht A. , TLR4-Based Immunotherapeutics in Cancer: A Review of the Achievements and Shortcomings, Molecular Pharmaceutics. (2018) 15, no. 11, 4777–4800, 10.1021/acs.molpharmaceut.8b00691, 2-s2.0-85054640204, 30226786. 30226786

[94] Pesce S. , Greppi M. , Ferretti E. , Obino V. , Carlomagno S. , Rutigliani M. , Thoren F. B. , Sivori S. , Castagnola P. , Candiani S. , and Marcenaro E. , miRNAs in NK Cell-Based Immune Responses and Cancer Immunotherapy, Frontiers in Cell and Developmental Biology. (2020) 8, 10.3389/fcell.2020.00119, 32161759. PMC7053181 32161759

[95] Zhao Y. , Qu Y. , Hao C. , and Yao W. , PD-1/PD-L1 Axis in Organ Fibrosis, Frontiers in Immunology. (2023) 14, 1145682, 10.3389/fimmu.2023.1145682, 37275876. 37275876 PMC10235450

[96] Li J. and Li A. , Role of microRNA 4717, its Effects on Programmed Cell Death Protein-1 in Hepatitis B Infection, and Interaction Between PDCD1 and miR-4717, European Journal of Inflammation. (2020) 18, 2058739220934604, 10.1177/2058739220934604.

[97] Colaianni F. , Zelli V. , Compagnoni C. , Miscione M. S. , Rossi M. , Vecchiotti D. , Di Padova M. , Alesse E. , Zazzeroni F. , and Tessitore A. , Role of Circulating microRNAs in Liver Disease and HCC: Focus on miR-122, Genes. (2024) 15, no. 10, 10.3390/genes15101313, 39457437. PMC11507253 39457437

[98] Mirzaei R. , Karampoor S. , and Korotkova N. L. , The Emerging Role of miRNA-122 in Infectious Diseases: Mechanisms and Potential Biomarkers, Pathology - Research and Practice. (2023) 249, 154725, 10.1016/j.prp.2023.154725, 37544130. 37544130

[99] Gelfo V. , Romaniello D. , Mazzeschi M. , Sgarzi M. , Grilli G. , Morselli A. , Manzan B. , Rihawi K. , and Lauriola M. , Roles of IL-1 in Cancer: From Tumor Progression to Resistance to Targeted Therapies, International Journal of Molecular Sciences. (2020) 21, no. 17, 10.3390/ijms21176009, 32825489. PMC7503335 32825489

[100] Liu Z.-L. , Chen H.-H. , Zheng L.-L. , Sun L.-P. , and Shi L. , Angiogenic Signaling Pathways and Anti-Angiogenic Therapy for Cancer, Signal Transduction and Targeted Therapy. (2023) 8, no. 1, 10.1038/s41392-023-01460-1. PMC10175505 37169756

[101] Ding J. , Gao Y. , He Y. , Zhou Y. , Huang M. , and Liu H. , Screening SNPs Residing in the microRNA-Binding Sites of Hepatocellular Carcinoma Related Genes, International Journal of Data Mining and Bioinformatics. (2011) 5, no. 1, 1–21, 10.1504/IJDMB.2011.038574, 2-s2.0-79951630825, 21491841. 21491841

[102] Chastney M. R. , Kaivola J. , Leppänen V.-M. , and Ivaska J. , The Role and Regulation of Integrins in Cell Migration and Invasion, Nature Reviews Molecular Cell Biology. (2025) 26, no. 2, 147–167, 10.1038/s41580-024-00777-1. 39349749

[103] Annese T. , Tamma R. , De Giorgis M. , and Ribatti D. , MicroRNAs Biogenesis, Functions and Role in Tumor Angiogenesis, Frontiers in Oncology. (2020) 10, 581007, 10.3389/fonc.2020.581007, 33330058. 33330058 PMC7729128

[104] Zheng Q. and Hou W. , Regulation of Angiogenesis by MicroRNAs in Cancer, Molecular Medicine Reports. (2021) 24, no. 2, 1–13, 10.3892/mmr.2021.12222, 34132365. PMC8223106 34132365

[105] Hamidi A. A. , Taghehchian N. , Basirat Z. , Zangouei A. S. , and Moghbeli M. , MicroRNAs as the Critical Regulators of Cell Migration and Invasion in Thyroid Cancer, Biomarker Research. (2022) 10, no. 1, 10.1186/s40364-022-00382-4, 35659780. PMC9167543 35659780

[106] Zhu Z. , Jiang Y. , Chen S. , Jia S. , Gao X. , Dong D. , and Gao Y. , An Insertion/Deletion Polymorphism in the 3’ Untranslated Region of Type I Collagen a2 (COL1A2) Is Associated With Susceptibility for Hepatocellular Carcinoma in a Chinese Population, Cancer Genetics. (2011) 204, no. 5, 265–269, 10.1016/j.cancergen.2011.03.007, 2-s2.0-80051586183, 21665180. 21665180

[107] Hua K.-T. , Liu Y.-F. , Hsu C.-L. , Cheng T.-Y. , Yang C.-Y. , Chang J.-S. , Lee W.-J. , Hsiao M. , Juan H.-F. , Chien M.-H. , and Yang S. F. , 3’UTR Polymorphisms of Carbonic Anhydrase IX Determine the miR-34a Targeting Efficiency and Prognosis of Hepatocellular Carcinoma, Scientific Reports. (2017) 7, no. 1, 10.1038/s41598-017-04732-3, 2-s2.0-85021675317, 28667334. PMC5493636 28667334

[108] Guo F. , Li H. , Wang L. , Song X. , Wang J. , Feng Q. , and Zong J. , Rs6757 in MicroRNA-3976 Binding Site of CD147 Confers Risk of Hepatocellular Carcinoma in South Chinese Population, World Journal of Surgical Oncology. (2022) 20, no. 1, 10.1186/s12957-022-02724-w, 35978360. PMC9382786 35978360

[109] Peng C. , Guo Z. , Wu X. , and Zhang X.-l. , A Polymorphism at the MicroRNA Binding Site in the 3’ Untranslated Region of RYR3 Is Associated With Outcome in Hepatocellular Carcinoma, Oncotargets and Therapy. (2015) 8, 2075–2079, 10.2147/OTT.S85856, 2-s2.0-84939789072, 26309413. 26309413 PMC4539090

[110] Chen X. , Zhang H. , Ou S. , and Chen H. , Von Hippel-Lindau Gene Single Nucleotide Polymorphism (rs1642742) May Be Related to the Occurrence and Metastasis of HBV-Related Hepatocellular Carcinoma, Medicine. (2021) 100, no. 35, e27187, 10.1097/MD.0000000000027187, 34477178. 34477178 PMC8415925

[111] Zhang H.-d. , Jiang L.-h. , Sun D.-w. , Li J. , and Ji Z.-l. , The Role of miR-130a in Cancer, Breast Cancer. (2017) 24, no. 4, 521–527, 10.1007/s12282-017-0776-x, 2-s2.0-85018769107. 28477068

[112] Mu H. , He Y. , Wang S. , Yang S. , Wang Y. , Nan C. , Bao Y. , Xie Q. , and Chen Y. , MiR-130b/TNF-*α*/NF-*κ*B/VEGFA Loop Inhibits Prostate Cancer Angiogenesis, Clinical and Translational Oncology. (2020) 22, no. 1, 111–121, 10.1007/s12094-019-02217-5, 31667686. 31667686

[113] Xie C. , Liu S. , Wu B. , Zhao Y. , Chen B. , Guo J. , Qiu S. , and Cao Y. M. , miR-19 Promotes Cell Proliferation, Invasion, Migration, and EMT by Inhibiting SPRED2-Mediated Autophagy in Osteosarcoma Cells, Cell Transplantation. (2020) 29, 963689720962460, 10.1177/0963689720962460, 33023313. 33023313 PMC7784565

[114] Li X. , Teng C. , Ma J. , Fu N. , Wang L. , Wen J. , and Wang T. Y. , miR-19 Family: A Promising Biomarker and Therapeutic Target in Heart, Vessels and Neurons, Life Sciences. (2019) 232, 116651, 10.1016/j.lfs.2019.116651, 2-s2.0-85068998811. 31302195

[115] Ji J. , Zhao L. , Budhu A. , Forgues M. , Jia H. L. , Qin L. X. , Ye Q. H. , Yu J. , Shi X. , Tang Z. Y. , and Wang X. W. , Let-7g Targets Collagen Type I Alpha2 and Inhibits Cell Migration in Hepatocellular Carcinoma, Journal of Hepatology. (2010) 52, no. 5, 690–697, 10.1016/j.jhep.2009.12.025, 2-s2.0-77951623294, 20338660. 20338660 PMC2862772

[116] Zhang Y. , Li Y. , Wang Q. , Su B. , Xu H. , Sun Y. , Sun P. , Li R. , Peng X. , and Cai J. , Role of RASA1 in Cancer: A Review and Update (Review), Oncology Reports. (2020) 44, no. 6, 2386–2396, 10.3892/or.2020.7807, 33125148. 33125148 PMC7610306

[117] Bernstein D. L. , Jiang X. , and Rom S. , Let-7 microRNAs: Their Role in Cerebral and Cardiovascular Diseases, Inflammation, Cancer, and their Regulation, Biomedicines. (2021) 9, no. 6, 10.3390/biomedicines9060606, 34073513. PMC8227213 34073513

[118] Yu X. , Huang J. , Liu X. , Li J. , Yu M. , Li M. , Xie Y. , Li Y. , Qiu J. , Xu Z. , Zhu T. , and Zhang W. , LncRNAH19 Acts as a ceRNA of Let-7 g to Facilitate Endothelial-to-Mesenchymal Transition in Hypoxic Pulmonary Hypertension via Regulating TGF-*β* Signalling Pathway, Respiratory Research. (2024) 25, no. 1, 10.1186/s12931-024-02895-y, 38987833. PMC11238495 38987833

[119] Li X. , Jin Y. , and Xue J. , Unveiling Collagen’s Role in Breast Cancer: Insights Into Expression Patterns, Functions and Clinical Implications, International Journal of General Medicine. (2024) 17, 1773–1787, 10.2147/IJGM.S463649, 38711825. 38711825 PMC11073151

[120] Li S. , Wei X. , He J. , Cao Q. , Du D. , Zhan X. , Zeng Y. , Yuan S. , and Sun L. , The Comprehensive Landscape of miR-34a in Cancer Research, Cancer Metastasis Reviews. (2021) 40, no. 3, 925–948, 10.1007/s10555-021-09973-3, 33959850. 33959850

[121] Li Q. and Zhang Q. , MiR-34a and Endothelial Biology, Life Sciences. (2023) 330, 121976, 10.1016/j.lfs.2023.121976. 37495076

[122] Zhang Y. , Ren H. , Li J. , Xue R. , Liu H. , Zhu Z. , Pan C. , Lin Y. , Hu A. , Gou P. , Cai J. , Zhou J. , Zhu W. , and Shi X. , Elevated HMGB1 Expression Induced by Hepatitis B Virus X Protein Promotes Epithelial-Mesenchymal Transition and Angiogenesis Through STAT3/miR-34a/NF-*κ*B in Primary Liver Cancer, American Journal of Cancer Research. (2021) 11, no. 2, 479–494, 33575082. 33575082 PMC7868754

[123] Niu X. , Wei N. , Peng L. , Li X. , Zhang X. , and Wang C. , miR-34a-5p Plays an Inhibitory Role in Hepatocellular Carcinoma by Regulating Target Gene VEGFA, Malaysian Journal of Pathology. (2022) 44, no. 1, 39–52, 35484885. 35484885

[124] Sun J. , Li Y. , Tian H. , Li J. , Shi M. , Chen S. , Guo Y. , La T. , and Li Z. , Long Non-coding RNA DDX11-AS1 Accelerates Hepatocellular Carcinoma Progression via Upregulating CA9 Expression and the MEK/ERK Pathway, HPB. (2023) 25, S390–S391, 10.1016/j.hpb.2023.07.400.

[125] Toole B. P. , The CD147-HYALURONAN Axis in Cancer, Anatomical Record. (2020) 303, no. 6, 1573–1583, 10.1002/ar.24147, 2-s2.0-85066914632, 31090215. 31090215

[126] Huang D. , Rao D. , Jin Q. , Lai M. , Zhang J. , Lai Z. , Shen H. , and Zhong T. , Role of CD147 in the Development and Diagnosis of Hepatocellular Carcinoma, Frontiers in Immunology. (2023) 14, 10.3389/fimmu.2023.1149931. PMC10115957 37090718

[127] Nyalali A. M. K. , Leonard A. U. , Xu Y. , Li H. , Zhou J. , Zhang X. , Rugambwa T. K. , Shi X. , and Li F. , CD147: An Integral and Potential Molecule to Abrogate Hallmarks of Cancer, Front Oncologia. (2023) 13, 1238051, 10.3389/fonc.2023.1238051, 38023152. PMC10662318 38023152

[128] Muniandy S. , Few L. L. , Khoo B. Y. , Hassan S. A. , Yvonne-Τee G. B. , and See Too W. C. , Dysregulated Expression of miR-367 in Disease Development and its Prospects as a Therapeutic Target and Diagnostic Biomarker, Biomedical Reports. (2023) 19, no. 6, 10.3892/br.2023.1673. PMC10603372 37901877

[129] Zhang L. , Liu Y. , Song F. , Zheng H. , Hu L. , Lu H. , Liu P. , Hao X. , Zhang W. , and Chen K. , Functional SNP in the MicroRNA-367 Binding Site in the 3’UTR of the Calcium Channel Ryanodine Receptor Gene 3 (RYR3) Affects Breast Cancer Risk and Calcification, Proceedings of the National Academy of Sciences of the United States of America. (2011) 108, no. 33, 13653–13658, 10.1073/pnas.1103360108, 2-s2.0-80052001024, 21810988. 21810988 PMC3158174

[130] Ruff S. E. , Logan S. K. , Garabedian M. J. , and Huang T. T. , Roles for MDC1 in Cancer Development and Treatment, DNA Repair. (2020) 95, 102948, 10.1016/j.dnarep.2020.102948, 32866776. 32866776 PMC7669677

[131] Akhlaghipour I. , Fanoodi A. , Zangouei A. S. , Taghehchian N. , Khalili-Tanha G. , and Moghbeli M. , MicroRNAs as the Critical Regulators of Forkhead Box Protein Family in Pancreatic, Thyroid, and Liver Cancers, Biochemical Genetics. (2023) 61, no. 5, 1645–1674, 10.1007/s10528-023-10346-4. 36781813

[132] Shojaei S. , Hashemi S. M. , Ghanbarian H. , Sharifi K. , Salehi M. , and Mohammadi-Yeganeh S. , Delivery of miR-381-3p Mimic by Mesenchymal Stem Cell-Derived Exosomes Inhibits Triple Negative Breast Cancer Aggressiveness; an In Vitro Study, Stem Cell Reviews and Reports. (2021) 17, no. 3, 1027–1038, 10.1007/s12015-020-10089-4, 33410095. 33410095

[133] Sha H. , Gan Y. , Xu F. , Zhu Y. , Zou R. , Peng W. , Wu Z. , Ma R. , Wu J. , and Feng J. , MicroRNA-381 in Human Cancer: Its Involvement in Tumour Biology and Clinical Applications Potential, Journal of Cellular and Molecular Medicine. (2022) 26, no. 4, 977–989, 10.1111/jcmm.17161. 35014178 PMC8831973

[134] Romero D. , Belzutifan Is Active in VHL-Related Cancers, Nature Reviews Clinical Oncology. (2022) 19, no. 2, 10.1038/s41571-021-00587-w, 34893759. 34893759

[135] Shah V. and Shah J. , Recent Trends in Targeting miRNAs for Cancer Therapy, Journal of Pharmacy and Pharmacology. (2020) 72, no. 12, 1732–1749, 10.1111/jphp.13351. 32783235

[136] Shao Y. , Shen Y. , Zhao L. , Guo X. , Niu C. , and Liu F. , Association of MicroRNA Biosynthesis Genes XPO5 and RAN Polymorphisms With Cancer Susceptibility: Bayesian Hierarchical Meta-Analysis, Journal of Cancer. (2020) 11, no. 8, 2181–2191, 10.7150/jca.37150. 32127945 PMC7052917

[137] Galka-Marciniak P. , Urbanek-Trzeciak M. O. , Nawrocka P. M. , and Kozlowski P. , A Pan-Cancer Atlas of Somatic Mutations in miRNA Biogenesis Genes, Nucleic Acids Research. (2021) 49, no. 2, 601–620, 10.1093/nar/gkaa1223, 33406242. 33406242 PMC7826265

[138] Dragomir M. P. , Knutsen E. , and Calin G. A. , Classical and Noncanonical Functions of miRNAs in Cancers, Trends in Genetics. (2022) 38, no. 4, 379–394, 10.1016/j.tig.2021.10.002, 34728089. 34728089

[139] Meng W. , Li Y. , Chai B. , Liu X. , and Ma Z. , miR-199a: A Tumor Suppressor with Noncoding RNA Network and Therapeutic Candidate in Lung Cancer, International Journal of Molecular Sciences. (2022) 23, no. 15, 10.3390/ijms23158518, 35955652. PMC9369015 35955652

[140] Liu L. , An J. , Liu J. , Wen J. , Zhai X. , Liu Y. , Pan S. , Jiang J. , Wen Y. , Liu Z. , Zhang Y. , Chen J. , Xing J. , Ji G. , Shen H. , Hu Z. , and Fan Z. , Potentially Functional Genetic Variants in microRNA Processing Genes and Risk of HBV-Related Hepatocellular Carcinoma, Molecular Carcinogenesis. (2013) 52, no. S1, 148–154, 10.1002/mc.22062, 2-s2.0-84886292273, 23868705. 23868705

[141] Li Y. , Zhang F. , and Xing C. , A Systematic Review and Meta-Analysis for the Association of Gene Polymorphisms in RAN with Cancer Risk, Disease Markers. (2020) 2020, 9026707, 10.1155/2020/9026707. 32015773 PMC6985935

[142] Yao C. , Ren J. , Huang R. , Tang C. , Cheng Y. , Lv Z. , Kong L. , Fang S. , Tao J. , Fu Y. , Zhu Q. , and Fang M. , Transcriptome Profiling of microRNAs Reveals Potential Mechanisms of Manual Therapy Alleviating Neuropathic Pain Through microRNA-547-3p-Mediated Map4k4/NF-*κ*b Signaling Pathway, Journal of Neuroinflammation. (2022) 19, no. 1, 10.1186/s12974-022-02568-x, 36045396. PMC9434879 36045396

[143] Vergani-Junior C. A. , Tonon-da-Silva G. , Inan M. D. , and Mori M. A. , DICER: Structure, Function, and Regulation, Biophysical Reviews. (2021) 13, no. 6, 1081–1090, 10.1007/s12551-021-00902-w, 35059029. 35059029 PMC8724510

[144] Li C. , Chen L. , Song W. , Peng B. , Zhu J. , and Fang L. , DICER Activates Autophagy and Promotes Cisplatin Resistance in Non-Small Cell Lung Cancer by Binding With Let-7i-5p, Acta Histochemica. (2021) 123, no. 7, 151788, 10.1016/j.acthis.2021.151788, 34543777. 34543777

[145] Kedmi M. , Sas-Chen A. , and Yarden Y. , MicroRNAs and Growth Factors: An Alliance Propelling Tumor Progression, Journal of Clinical Medicine. (2015) 4, no. 8, 1578–1599, 10.3390/jcm4081578, 26287249. 26287249 PMC4555078

[146] Hussen B. M. , Hidayat H. J. , Salihi A. , Sabir D. K. , Taheri M. , and Ghafouri-Fard S. , MicroRNA: A Signature for Cancer Progression, Biomedicine & Pharmacotherapy. (2021) 138, 111528, 10.1016/j.biopha.2021.111528. 33770669

[147] Orso F. , Quirico L. , Dettori D. , Coppo R. , Virga F. , Ferreira L. C. , Paoletti C. , Baruffaldi D. , Penna E. , and Taverna D. , Role of miRNAs in Tumor and Endothelial Cell Interactions During Tumor Progression, Seminars in Cancer Biology. (2020) 60, 214–224, 10.1016/j.semcancer.2019.07.024, 2-s2.0-85072220219. 31386907

[148] Gramantieri L. , Giovannini C. , Piscaglia F. , and Fornari F. , MicroRNAs as Modulators of Tumor Metabolism, Microenvironment, and Immune Response in Hepatocellular Carcinoma, Journal of Hepatocellular Carcinoma. (2021) 8, 369–385, 10.2147/jhc.S268292, 34012928. 34012928 PMC8126872

[149] Cantile M. , Di Bonito M. , Tracey De Bellis M. , and Botti G. , Functional Interaction Among lncRNA HOTAIR and MicroRNAs in Cancer and Other Human Diseases, Cancers. (2021) 13, no. 3, 10.3390/cancers13030570, 33540611. PMC7867281 33540611

[150] Yu Q. , Zhou C.-X. , Chen N.-S. , Zheng S.-D. , Shen L.-M. , and Zhang J.-K. , A Polymorphism Within ErbB4is Associated With Risk for Hepatocellular Carcinoma in Chinese Population, World Journal of Gastroenterology: WJG. (2012) 18, no. 4, 383–387, 10.3748/wjg.v18.i4.383, 2-s2.0-84862920049, 22294845. 22294845 PMC3261534

[151] Guo Z. , Wu C. , Wang X. , Wang C. , Zhang R. , and Shan B. , A Polymorphism at the miR-502 Binding Site in the 3’-Untranslated Region of the Histone methyltransferaseSET8is Associated With Hepatocellular Carcinoma Outcome, International Journal of Cancer. (2012) 131, no. 6, 1318–1322, 10.1002/ijc.27352, 2-s2.0-84860346238, 22095217. 22095217

[152] Fan Y. , Qian X. , and Zhang C. , U/G SNP rs111904020 in 3’ UTR of STAT3 Regulated by miR-214 Promotes Hepatocellular Carcinoma Development in Chinese Population, Tumor Biology. (2016) 37, no. 11, 14629–14635, 10.1007/s13277-016-5352-z, 2-s2.0-84987617371, 27619679. 27619679

[153] Fan Y. , Zhang C. , and Qian X. , Association of Polymorphisms in the 3’UTR of ErbB2 With the Risk of Hepatocellular Carcinoma, International Journal of Clinical & Experimental Medicine. (2016) 9, no. 2.

[154] Zhang L. , Li X. , Lu J. , Qian Y. , Qian T. , Wu X. , and Xu Q. , The egfr Polymorphism Increased the Risk of Hepatocellular Carcinoma Through the mir-3196-Dependent Approach in Chinese Han Population, Pharmacogenomics and Personalized Medicine. (2021) 14, 469–476, 10.2147/PGPM.S304524, 33935511. 33935511 PMC8079348

[155] Yu Q. , Qian W. , Wang J. , Wu Y. , Zhang J. , and Chen W. , An Indel Polymorphism in the 3′ Untranslated Region of JAK1 Confers Risk for Hepatocellular Carcinoma Possibly by Regulating JAK1 Transcriptional Activity in a Chinese Population, Oncology Letters. (2018) 15, no. 5, 8088–8094, 10.3892/ol.2018.8347, 2-s2.0-85044650269, 29731916. 29731916 PMC5921256

[156] Xiong G. , Wang Y. , Ding Q. , and Yang L. , Hsa-Mir-1269 Genetic Variant Contributes to Hepatocellular Carcinoma Susceptibility Through Affecting SOX6, American Journal of Translational Research. (2015) 7, no. 10, 2091–2098, 26692953. 26692953 PMC4656786

[157] Wang C. , Zhao H. , Zhao X. , Wan J. , Wang D. , Bi W. , Jiang X. , and Gao Y. , Association Between an Insertion/Deletion Polymorphism Within 3’ UTR of SGSM3 and Risk of Hepatocellular Carcinoma, Tumor Biology. (2014) 35, no. 1, 295–301, 10.1007/s13277-013-1039-x, 2-s2.0-84893764236, 23918301. 23918301

[158] Ma Y. , Shen N. , Wicha M. S. , and Luo M. , The Roles of the Let-7 Family of MicroRNAs in the Regulation of Cancer Stemness, Cells. (2021) 10, no. 9, 10.3390/cells10092415. PMC8469079 34572067

[159] Han S. , Zhang T. , Kusumanchi P. , Huda N. , Jiang Y. , Liangpunsakul S. , and Yang Z. , Role of microRNA-7 in Liver Diseases: A Comprehensive Review of the Mechanisms and Therapeutic Applications, Journal of Investigative Medicine. (2020) 68, no. 7, 1208–1216, 10.1136/jim-2020-001420. 32843369 PMC9303053

[160] El-Gamal M. I. , Mewafi N. H. , Abdelmotteleb N. E. , Emara M. A. , Tarazi H. , Sbenati R. M. , Madkour M. M. , Zaraei S. O. , Shahin A. I. , and Anbar H. S. , A Review of HER4 (ErbB4) Kinase, Its Impact on Cancer, and Its Inhibitors, Molecules. (2021) 26, no. 23, 10.3390/molecules26237376, 34885957. PMC8659013 34885957

[161] Qi W. , Gao C. , Zhang L. , Gao Z. , Sui J. , Han C. , and Sun D. , MiR-3196, a p53-Responsive microRNA, Functions as a Tumor Suppressor in Hepatocellular Carcinoma by Targeting FOXP4, American Journal of Cancer Research. (2019) 9, no. 12, 2665–2678, 31911853. 31911853 PMC6943357

[162] Jun H. , Jang E. , Kim H. , Yeo M. , Park S. G. , Lee J. , Shin K. J. , Chae Y. C. , Kang S. , and Kim E. , TRAIL & EGFR Affibody Dual-Display on a Protein Nanoparticle Synergistically Suppresses Tumor Growth, Journal of Controlled Release. (2022) 349, 367–378, 10.1016/j.jconrel.2022.07.004. 35809662

[163] Uribe M. L. , Marrocco I. , and Yarden Y. , EGFR in Cancer: Signaling Mechanisms, Drugs, and Acquired Resistance, Cancers. (2021) 13, no. 11, 10.3390/cancers13112748. PMC8197917 34206026

[164] Gao J. , Huo Z. , Song X. , Shao Q. , Ren W. , Huang X. , Zhou S. , and Tang X. , EGFR Mediates Epithelial-Mesenchymal Transition Through the Akt/GSK-3*β*/Snail Signaling Pathway to Promote Liver Cancer Proliferation and Migration, Oncology Letters. (2023) 27, no. 2, 10.3892/ol.2023.14192, 38192662. PMC10773224 38192662

[165] Sukmana B. I. , Al-Hawary S. I. S. , Abosaooda M. , Adile M. , Gupta R. , Saleh E. A. M. , Alwaily E. R. , Alsaab H. O. , Sapaev I. B. , and Mustafa Y. F. , A Thorough and Current Study of miR-214-Related Targets in Cancer, Pathology, Research and Practice. (2023) 249, 154770, 10.1016/j.prp.2023.154770, 37660658. 37660658

[166] Sagar S. K. , miR-106b as an Emerging Therapeutic Target in Cancer, Genes & Diseases. (2022) 9, no. 4, 889–899, 10.1016/j.gendis.2021.02.002, 35685464. 35685464 PMC9170583

[167] Karimi E. , Dehghani A. , Azari H. , Zarei M. , Shekari M. , and Mousavi P. , Molecular Mechanisms of miR-214 Involved in Cancer and Drug Resistance, Current Molecular Medicine. (2023) 23, no. 7, 589–605, 10.2174/1566524022666220428112744, 37282586. 37282586

[168] Gargalionis A. N. , Papavassiliou K. A. , and Papavassiliou A. G. , Targeting STAT3 Signaling Pathway in Colorectal Cancer, Biomedicine. (2021) 9, no. 8, 10.3390/biomedicines9081016. PMC8392110 34440220

[169] Hin Tang J. J. , Hao Thng D. K. , Lim J. J. , and Toh T. B. , JAK/STAT Signaling in Hepatocellular Carcinoma, Hepatic Oncology. (2020) 7, no. 1, HEP18, 10.2217/hep-2020-0001. 32273976 PMC7137178

[170] Di Martino M. T. , Arbitrio M. , Caracciolo D. , Cordua A. , Cuomo O. , Grillone K. , Riillo C. , Caridà G. , Scionti F. , and Labanca C. , miR-221/222 as Biomarkers and Targets for Therapeutic Intervention on Cancer and Other Diseases: A Systematic Review, Molecular Therapy Nucleic Acids. (2022) 27, 1191–1224, 10.1016/j.omtn.2022.02.005, 35282417. 35282417 PMC8891816

[171] Liu W. , Hu K. , Zhang F. , Lu S. , Chen R. , Ren Z. , and Yin X. , The Prognostic Significance of microRNA-221 in Hepatocellular Carcinoma: An Updated Meta-Analysis, International Journal of Biological Markers. (2021) 36, no. 2, 17246008211032689, 10.1177/17246008211032689, 34374576. 34374576

[172] Chang W. , Chang Q. , Lu H. , Li Y. , and Chen C. , Mir-221-3p Facilitates Thyroid Cancer Cell Proliferation and Inhibit Apoptosis by Targeting FOXP2 Through Hedgehog Pathway, Molecular Biotechnology. (2022) 64, no. 8, 919–927, 10.1007/s12033-022-00473-5, 35257310. 35257310

[173] Döring P. , Calvisi D. F. , and Dombrowski F. , Nuclear ErbB2 Expression in Hepatocytes in Liver Disease, Virchows Archiv. (2021) 478, no. 2, 309–318, 10.1007/s00428-020-02871-z, 32591879. 32591879 PMC7969555

[174] You H. , Yuan D. , Li Q. , Zhang N. , Kong D. , Yu T. , Liu X. , Liu X. , Zhou R. , Kong F. , Zheng K. , and Tang R. , Hepatitis B Virus X Protein Increases LASP1 SUMOylation to Stabilize HER2 and Facilitate Hepatocarcinogenesis, International Journal of Biological Macromolecules. (2023) 226, 996–1009, 10.1016/j.ijbiomac.2022.11.312, 36473530. 36473530

[175] You W. , Liu X. , Yu Y. , Chen C. , Xiong Y. , Liu Y. , Sun Y. , Tan C. , Zhang H. , Wang Y. , and Li R. , miR-502-5p Affects Gastric Cancer Progression by Targeting PD-L1, Cancer Cell International. (2020) 20, no. 1, 1–13, 10.1186/s12935-020-01479-2. 32821248 PMC7429713

[176] Qi J. , Chen X. , Wu Q. , Wang J. , Zhang H. , Mao A. , Zhu M. , and Miao C. , Fasting Induces Hepatocellular Carcinoma Cell Apoptosis by Inhibiting SET8 Expression, Oxidative Medicine and Cellular Longevity. (2020) 2020, 3985089, 10.1155/2020/3985089. 32273943 PMC7115168

[177] Wu J. , Qiao K. , Du Y. , Zhang X. , Cheng H. , Peng L. , and Guo Z. , Downregulation of histone methyltransferase _SET8_ inhibits progression of hepatocellular carcinoma, Scientific Reports. (2020) 10, no. 1, 10.1038/s41598-020-61402-7, 32161353. PMC7066161 32161353

[178] Dong H. , Huang C. , and Huang J. , FBXL19-AS1 promotes the Progression of Nasopharyngeal Carcinoma by Acting AS a Competing Endogenous RNA to Sponge miR-431 and Upregulate PBOV1, Molecular Medicine Reports. (2021) 24, no. 3, 10.3892/mmr.2021.12286, 34278444. PMC8299196 34278444

[179] Wu W. , Zhou Z. , Chen C. , and Chen M. , Circ_0061395 Functions as an Oncogenic Gene in Hepatocellular Carcinoma by Acting as a miR-1182 Sponge, Cell Cycle. (2022) 21, no. 20, 2192–2205, 10.1080/15384101.2022.2092177, 35775884. 35775884 PMC9519000

[180] Park H. , Lee S. , Lee J. , Moon H. , and Ro S. W. , Exploring the JAK/STAT Signaling Pathway in Hepatocellular Carcinoma: Unraveling Signaling Complexity and Therapeutic Implications, International Journal of Molecular Sciences. (2023) 24, no. 18, 10.3390/ijms241813764, 13764, 37762066. 37762066 PMC10531214

[181] Ni L. , Wang X. , Gu J. , Hao S. , and Sun L. , Total Flavones of *Selaginella uncinata* (Desv.) Spring Inhibits Breast Cancer Cell Proliferation and Induces Apoptosis via Regulating microRNA-1269, Indian Journal of Pharmaceutical Sciences. (2023) 85, no. 3, 10.36468/pharmaceutical-sciences.1149.

[182] Xie Z. , Zhong C. , and Duan S. , miR-1269a And miR-1269b: Emerging Carcinogenic Genes of the miR-1269 Family, Frontiers in Cell and Developmental Biology. (2022) 10, 809132, 10.3389/fcell.2022.809132, 35252180. 35252180 PMC8894702

[183] Zhou C. , Hu C. , Wang B. , Fan S. , and Jin W. , Curcumin Suppresses Cell Proliferation, Migration, and Invasion Through Modulating miR-21-5p/SOX6Axis in Hepatocellular Carcinoma, Cancer Biotherapy and Radiopharmaceuticals, 2020, Mary Ann Liebert, Inc., 10.1089/cbr.2020.3734. 32757994

[184] Pastori V. , Zambanini G. , Citterio E. , Weiss T. , Nakamura Y. , Cantù C. , and Ronchi A. E. , Transcriptional Repression of the Oncofetal *LIN28B* Gene by the Transcription Factor SOX6, Scientific Reports. (2024) 14, no. 1, 10287, 10.1038/s41598-024-60438-3, 38704454. 38704454 PMC11069503

[185] Yue C. , Chen X. , Li J. , Yang X. , Li Y. , and Wen Y. , miR-151-3p Inhibits Proliferation and Invasion of Colon Cancer Cell by Targeting Close Homolog of L1, Journal of Biomedical Nanotechnology. (2020) 16, no. 6, 876–884, 10.1166/jbn.2020.2941, 33187583. 33187583

[186] Guo Z. , Shu Y. , Zhou H. , and Zhang W. , Identification of Diagnostic and Prognostic Biomarkers for Cancer: Focusing on Genetic Variations in MicroRNA Regulatory Pathways (Review), Molecular Medicine Reports. (2016) 13, no. 3, 1943–1952, 10.3892/mmr.2016.4782, 2-s2.0-84958719171, 26782081. 26782081

[187] Jin J. and Ren M. , The Biological Function of miR-128-2 in Hepatocellular Carcinoma and Its Molecular Mechanism Functioning, Pathology-Research and Practice. (2024) 254, 155178, 10.1016/j.prp.2024.155178, 38309020. 38309020

[188] Zhuang L. , Xu L. , Wang P. , and Meng Z. , Serum miR-128-2 Serves as a Prognostic Marker for Patients With Hepatocellular Carcinoma, PLoS One. (2015) 10, no. 2, e0117274, 10.1371/journal.pone.0117274, 2-s2.0-84922246972, 25642945. 25642945 PMC4313939

[189] Yu D. , Green B. , Marrone A. , Guo Y. , Kadlubar S. , Lin D. , Fuscoe J. , Pogribny I. , and Ning B. , Suppression of CYP2C9 by microRNA hsa-miR-128-3p in Human Liver Cells and Association With Hepatocellular Carcinoma, Scientific Reports. (2015) 5, no. 1, 10.1038/srep08534, 2-s2.0-84923374447. PMC4336941 25704921

[190] Jiang Z. , Zheng X. , Wang W. , Qiu L. , Yang L. , Jiang M. , and Hua Y. , CYP2C9 Inhibits the Invasion and Migration of Esophageal Squamous Cell Carcinoma Via Downregulation of HDAC, Molecular and Cellular Biochemistry. (2021) 476, no. 5, 2011–2020, 10.1007/s11010-021-04050-3, 33515198. 33515198

[191] Uthansingh K. , Parida P. K. , Pati G. K. , Sahu M. K. , and Padhy R. N. , Evaluating the Association of Genetic Polymorphism of Cytochrome p450 (CYP2C9∗3) in Gastric Cancer Using Polymerase Chain Reaction-Restriction Fragment Length Polymorphism (PCR-RFLP), Cureus. (2022) 14, no. 7, e27220, 10.7759/cureus.27220, 36035062. 36035062 PMC9399687

